# Checkpoint inhibition of origin firing prevents DNA topological stress

**DOI:** 10.1101/gad.328682.119

**Published:** 2019-11-01

**Authors:** Esther C. Morafraile, Christine Hänni, George Allen, Theresa Zeisner, Caroline Clarke, Mark C. Johnson, Miguel M. Santos, Lauren Carroll, Nicola E. Minchell, Jonathan Baxter, Peter Banks, Dave Lydall, Philip Zegerman

**Affiliations:** 1 Wellcome Trust/Cancer Research UK Gurdon Institute and Department of Biochemistry, University of Cambridge CB2 1QN, United Kingdom;; 2 Genome Damage and Stability Centre, University of Sussex, Falmer, Brighton, East Sussex BN1 9RQ, United Kingdom;; 3 Institute for Cell and Molecular Biosciences, The Medical School, Newcastle University, Newcastle upon Tyne NE2 4HH, United Kingdom

**Keywords:** checkpoint, DNA topology, genome stability, origin firing, Rad53, S-phase, supercoiling, catenation, yeast

## Abstract

A universal feature of DNA damage and replication stress in eukaryotes is the activation of a checkpoint-kinase response. In S-phase, the checkpoint inhibits replication initiation, yet the function of this global block to origin firing remains unknown. To establish the physiological roles of this arm of the checkpoint, we analyzed separation of function mutants in the budding yeast *Saccharomyces cerevisiae* that allow global origin firing upon replication stress, despite an otherwise normal checkpoint response. Using genetic screens, we show that lack of the checkpoint-block to origin firing results in a dependence on pathways required for the resolution of topological problems. Failure to inhibit replication initiation indeed causes increased DNA catenation, resulting in DNA damage and chromosome loss. We further show that such topological stress is not only a consequence of a failed checkpoint response but also occurs in an unperturbed S-phase when too many origins fire simultaneously. Together we reveal that the role of limiting the number of replication initiation events is to prevent DNA topological problems, which may be relevant for the treatment of cancer with both topoisomerase and checkpoint inhibitors.

## Abstract (precis)

In this study, Morafraile et al. set out to understand why replication origin activation is blocked upon activation of the intra-S-phase checkpoint in response to replication stress. Using genetic screens and separation of function mutants in S. cerevisiae, the authors show that limiting the number of replication initiation events prevents DNA topological problems that lead to DNA damage and chromosome loss, thus providing new insights into the physiological importance of limiting the total number of replication initiation events in response to DNA damage.

To ensure the timely and complete duplication of the genome, eukaryotic chromosomes are replicated from multiple origins. As a result, eukaryotic replication must be strictly regulated so that no origin fires more than once per S-phase. This is achieved by close linkage between replication initiation and cell cycle control (Bell and Labib 2016). The first step in replication (prereplicative complex assembly or “licensing”) involves the loading of inactive double hexamers of the Mcm2-7 helicase at origins in G1 phase. Initiation at these origins can only occur in S-phase due to the activation of the S-phase CDK (S-CDK) and Dbf4-dependent (DDK) kinases. DDK directly phosphorylates Mcm2-7 double hexamers, while CDK phosphorylates two essential initiation factors, Sld3 and Sld2. Together, DDK and CDK are required for the assembly of the active replicative helicase and for the recruitment of additional proteins to form the multi-subunit replication machinery, called the replisome.

Although S-CDK and DDK both accumulate at the G1-S transition, origins do not all fire simultaneously but instead fire throughout S-phase (Rhind and Gilbert 2013). The timing of firing of an origin is stereotypical, with some origins more likely to fire early in S-phase, some in late S-phase, while others do not fire at all in a normal S-phase, so-called dormant origins (McIntosh and Blow 2012). Origin firing time is affected by several factors, including the chromatin environment and subsequent accessibility to limiting replication initiation factors, which include CDK targets Sld2 and Sld3, as well as the DDK subunit Dbf4 (Mantiero et al. 2011; Tanaka et al. 2011). A temporal order of origin firing, together with dormant origins, likely acts as a back-up mechanism to ensure complete genome duplication even if irreparable damage occurs at one or more replication forks (McIntosh and Blow 2012).

Replication stress, for example, caused by DNA lesions, conflicts between DNA and RNA polymerase, or low levels of deoxynucleotide triphosphates (dNTPs), is an early event during tumorigenesis (Kotsantis et al. 2018). Such stress leads to stalling of the replisome and activation of the checkpoint kinase ATR/Mec1, which causes the subsequent activation of the effector kinase Chk1 in humans or Rad53 in yeast (Giannattasio and Branzei 2017). This response to replication stress is called the S-phase, intra-S-phase, or DNA replication checkpoint (Pardo et al. 2017).

The S-phase checkpoint results in a range of responses including the up-regulation of dNTPs, DNA repair, and fork stabilization, which enables forks to resume replication after stalling (Giannattasio and Branzei 2017). In addition, it was observed over 40 years ago that DNA damage results in the inhibition of replication initiation (Painter 1977), which is checkpoint-dependent (Painter and Young 1980). Although the firing of local dormant origins allows stalled replication forks to be rescued, the checkpoint induces a global inhibition of replication initiation, resulting in the overall slowing of DNA synthesis in response to damage (Painter 1977; Paulovich and Hartwell 1995; McIntosh and Blow 2012). The function of this global inhibition of origin firing has remained unclear.

The mechanism of inhibition of origin firing by the checkpoint has been established in budding yeast (Lopez-Mosqueda et al. 2010; Zegerman and Diffley 2010). In response to DNA damage or fork stalling agents, the checkpoint kinase Rad53 phosphorylates and inhibits two replication initiation factors, Dbf4 and Sld3. These two substrates are the minimum targets for the checkpoint-dependent block to origin firing, because mutation of the Rad53-phosphorylated residues in Dbf4 and Sld3 allows replication initiation even when Rad53 is fully active (Lopez-Mosqueda et al. 2010; Zegerman and Diffley 2010). Although it is not clear how Rad53 inhibits Dbf4, phosphorylation of Sld3 by Rad53 prevents its interactions with other replication factors, including Dpb11 and Cdc45 (Lopez-Mosqueda et al. 2010; Zegerman and Diffley 2010). The checkpoint inhibition of origin firing is conserved across eukaryotes, and there are significant similarities in the mechanism of this control in metazoa, including the checkpoint inhibition of the Sld3 ortholog Treslin (Guo et al. 2015) and inhibition of DDK (Costanzo et al. 2003).

Here we take advantage of the separation of function alleles of *SLD3* and *DBF4* that cannot be inhibited by Rad53 (Zegerman and Diffley 2010) to analyze the role of the global inhibition of origin firing after replication stress in the budding yeast *Saccharomyces cerevisiae*. We show that a critical consequence of loss of the checkpoint block to initiation is the excessive DNA topological stress generated by large numbers of replication forks, resulting in DNA damage and chromosome loss. This study provides the first analysis of the role of this checkpoint response in isolation, which has implications for why most cells utilize only a fraction of their origins in a normal S-phase.

## Results

### The replication checkpoint inhibits origin firing genome-wide

**Figure 1.**
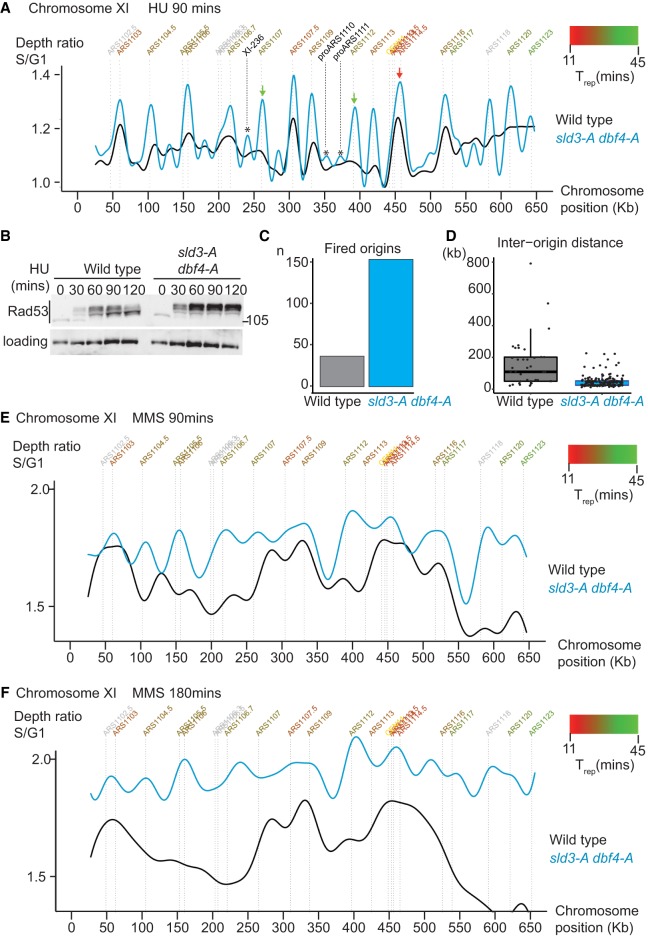
The replication checkpoint inhibits origin firing genome-wide. (*A*) Replication profile of the indicated strains after release from alpha factor into 200 mM HU for 90 min. Copy number (*y*-axis) was derived by normalizing the sequencing reads at 90 min to the reads at 0 min (G1). Annotated origins (*top*) are colored according to their average time of replication in an unperturbed S-phase (T_rep_) from early (red) to late (green). Only chromosome XI is shown for simplicity. Unconfirmed origins also fire in the *sld3-A dbf4-A* strain, examples of which are indicated by the *. The telomeres are excluded due to mappability issues. (*B*) Rad53 western blot from the indicated strains released from G1 phase arrest with alpha factor (0 min) into 200 mM HU for the indicated time points. (*C*) Graph of number of origins from *A* that fired in at least 20% of cells. (*D*) Box plot of each origin from *C* plotted according to the distance to its nearest neighboring fired origin. (*E*,*F*) As in *A*, except the strains were released from G1 phase into 0.02% MMS for 90 min (*E*) and 180 min (*F*).

Previously, we generated alleles of *SLD3* and *DBF4* in budding yeast that cannot be phosphorylated by the checkpoint kinase Rad53 (Zegerman and Diffley 2010). These alleles contain serine/threonine to alanine mutations at 38 sites in Sld3 and four sites in Dbf4 and are hereafter referred to as *sld3-A* and *dbf4-A*. These alleles are effective separation of function mutants because they are fully competent for their essential functions in replication initiation, yet they prevent checkpoint inhibition of origin firing while checkpoint activation and other functions of this pathway remain unaffected (Fig. 1B; Supplemental Fig. S1A; Zegerman and Diffley 2010).

To demonstrate the importance of checkpoint inhibition of origin firing genome-wide, we analyzed the replication dynamics of the *sld3-A dbf4-A* strain during replication stress by high-throughput sequencing. Replication profiles were obtained by comparing the DNA content of cells in G1 phase (arrested with the mating pheromone alpha factor) with those arrested in hydroxyurea (HU) after release from G1. A representative chromosome (Chr XI) from this analysis shows that wild-type cells (black line, Fig. 1A) initiate replication at early firing origins but not at late firing origins, as expected due to the activation of the checkpoint (Fig. 1B). Importantly, in the *sld3-A dbf4-A* mutant strain (blue line, Fig. 1A), not only did early origins fire efficiently, e.g., ARS1114.5 (red arrow, Fig. 1A), so did almost all other annotated origins (e.g., green arrows, Fig. 1A). Indeed, unannotated origins (see Siow et al. 2012) also fire in the *sld3-A dbf4-A* strain (indicated by [*] in Fig. 1A), including XI-236 and proARS1110 and proARS1111, consistent with a global effect of the checkpoint on origin firing. Early origins, such as ARS1114.5 (red arrow, Fig. 1A), appear to fire even more efficiently in the *sld3-A dbf4-A* strain, likely because the timing of origin firing (T_rep_) is an average, and in some wild-type cells, this origin is inhibited by the checkpoint. Despite this, the increase in origin firing in the *sld3-A dbf4-A* strain was greatest at late firing origins (Fig. 1A; Supplemental Fig. S1C), as expected (Zegerman and Diffley 2010).

Genome-wide analysis showed that over four times more origins fired in the *sld3-A dbf4-A* strain in HU (Fig. 1C), resulting in a greatly reduced interorigin distance (Fig. 1D). The *sld3-A dbf4-A* strain also displays greater Rad53 activation than a wild-type strain (Fig. 1B; Zegerman and Diffley 2010). Since Rad53 activation is proportional to the number of stalled forks (Tercero et al. 2003), this increased Rad53 activation is likely due to the greater number of forks in the *sld3-A dbf4-A* strain in HU (Fig. 1A). In addition, the peaks of replication in the *sld3-A dbf4-A* strain were narrower on average than in a wild-type strain (Supplemental Fig. S1D), suggesting that although more origins fire in this strain in HU, forks travel less far. This is consistent with previous studies showing that increased origin firing results in reduced fork progression, which in HU is likely due to the limiting pools of dNTPs (Poli et al. 2012; Zhong et al. 2013).

We have previously shown that the *sld3-A dbf4-A* strain has a fast S-phase in the presence of the DNA alkylating agent MMS (Zegerman and Diffley 2010). By performing a similar analysis as in HU, we now show that this fast S-phase in high doses of MMS is indeed due to a much greater degree of origin firing in the *sld3-A dbf4-A* strain at 90 min (Fig. 1E), resulting in near completion of S-phase by 180 min (Fig. 1F; Supplemental Fig. S1E). Together, these analyses show that the *sld3-A dbf4-A* alleles are excellent tools to analyze specifically the global inhibition of origin firing by the checkpoint.

### Checkpoint inhibition of origin firing prevents the accumulation of DNA damage markers

**Figure 2.**
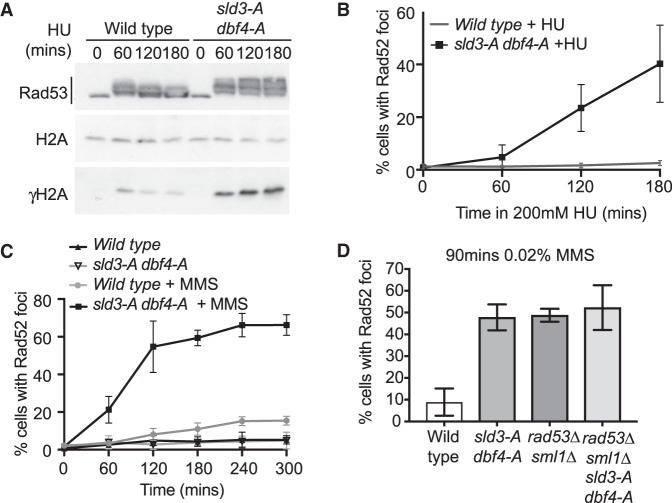
Checkpoint inhibition of origin firing prevents the accumulation of DNA damage markers. (*A*) Western blots from the indicated strains released from G1 phase arrest with alpha factor (0 min) into 200 mM HU for the indicated time points. (*B*) Quantification of Rad52-GFP foci in the indicated strains released from G1 phase arrest with alpha factor (0 min) into 200 mM HU for the indicated time points. Error bars are SD, *n* = 7. (*C*) As in *B*, except strains were released from G1 phase (0 min) into either 0.02% MMS or into medium in the absence of drug for the indicated time points. Error bars are SD, *n* = 5. (*D*) Quantification of Rad52-GFP foci in the indicated strains released from G1 phase arrest with alpha factor (0 min) into 0.02% MMS for 90 min. Error bars are SD, *n* = 3.

As the failure of the checkpoint inhibition of origin firing led to a dramatic increase in replication initiation (Fig. 1), we wondered whether this might result in genome instability. To address this, we analyzed the appearance of markers of DNA damage in the *sld3-A dbf4-A* strain. Checkpoint kinase-mediated phosphorylation of H2A at serine 129 resulting in γH2A (equivalent to metazoan γ-H2AX) is an early response to DNA damage and fork stalling (Szilard et al. 2010; Allen et al. 2011). Analysis of γH2A by western blot revealed that the *sld3-A dbf4-A* mutant strain has higher levels of γH2A than wild type in both HU and MMS, indicative of DNA damage (Fig. 2A; Supplemental Fig. S2A).

To further detect DNA damage accumulation, we analyzed the formation Rad52 foci. Rad52 is essential for double-strand break (DSB) repair through homologous recombination (HR) and forms foci at DSBs but also forms foci in response to fork stalling (Lisby et al. 2001, 2004; Allen et al. 2011). While we observed very little Rad52 foci formation in wild-type cells, there was a dramatic increase in Rad52 foci in the *sld3-A dbf4-A* strain in the presence of both HU and MMS (Fig. 2B,C), consistent with a previous study (Lopez-Mosqueda et al. 2010). These Rad52 foci were specific to replication stress in S-phase and were not suppressed by inhibiting mitosis or by increasing nucleotide concentrations (Supplemental Fig. S2B–D). Although γH2A and Rad52 foci occur at DSBs, the timing of accumulation of Rad52 foci in HU was coincident with S-phase progression (Supplemental Fig. S2E). This is consistent with previous reports showing that Rad52 foci can form due to replication stress independently of DSBs (Lisby et al. 2004; Szilard et al. 2010; Allen et al. 2011).

Checkpoint defective strains, such as *RAD53* null mutants, have been previously shown to accumulate Rad52 foci after fork stalling (Lisby et al. 2004). Significantly, a comparison between *sld3-A dbf4-A* and *rad53Δ* cells shows that the failure to inhibit origin firing accounts for the majority of Rad52 foci in this checkpoint mutant (Fig. 2D). Significantly, the *sld3-A dbf4-A* and *rad53*Δ alleles are epistatic for the formation of Rad52 foci (Fig. 2D), consistent with these mutants generating Rad52 foci by the same mechanism. From these data we conclude that the checkpoint-dependent inhibition of origin firing is an important pathway to prevent DNA damage marker accumulation in S-phase.

### Checkpoint inhibition of origin firing prevents DNA damage globally, but in particular at convergently transcribed genes

**Figure 3.**
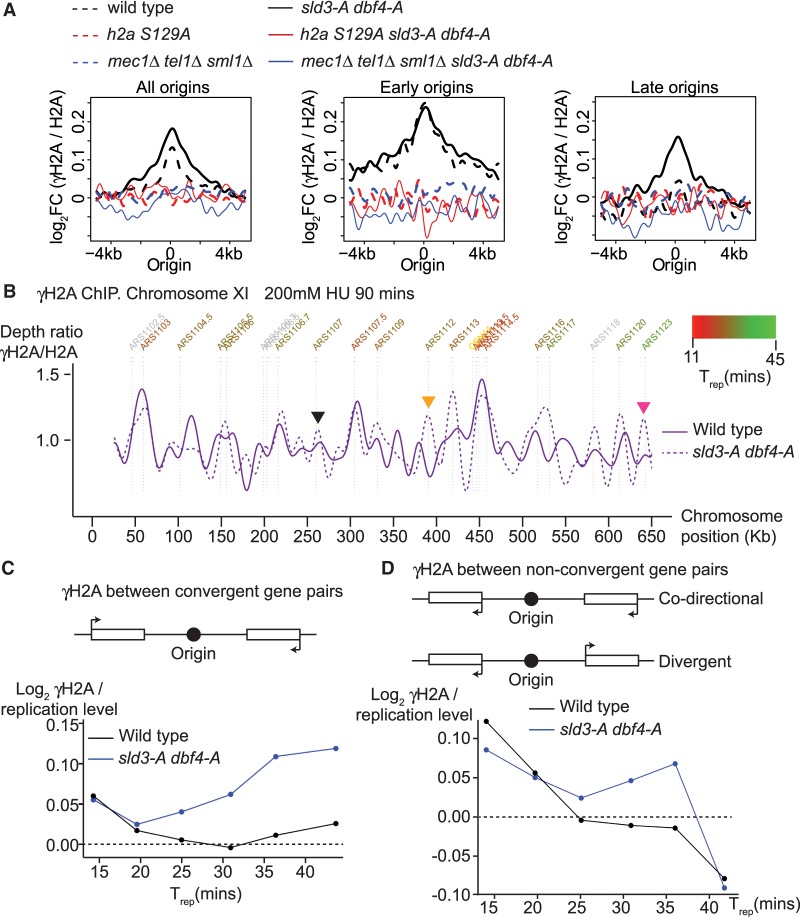
Checkpoint inhibition of origin firing prevents DNA damage globally but in particular at convergently transcribed gene pairs. (*A*) Graphs of γH2A ChIP from the indicated strains released from G1 phase arrest into 200 mM HU for 90 min. The graphs are the average γH2A ChIP signal centered on all origins (*left*), or origins split into early firing (*T*_rep_ < 27.5 min, *middle*), or late firing (*T*_rep_ > 27.5 min, *right*). Data are normalized to the ChIP signal of unmodified H2A. (*B*) Chromosomal view of data from *A*; only chromosome XI is shown for simplicity. Orange and black arrows indicate origins that fire efficiently in the *sld3-A dbf4-A* strain, while the pink arrow indicates an origin that does not fire efficiently in the *sld3-A dbf4-A* strain. (*C*,*D*) Schematic diagram of origins between convergently transcribed gene pairs (*C*, *top*), or between codirectional/divergent gene pairs (*D*, *top*). (*Bottom*) γH2A ChIP signal from *A* was normalized to the amount of replication at that locus. These data were binned according to average time of replication in a normal S-phase (T_rep_) and separated into those origins that are between convergently transcribed gene pairs (*C*) or nonconvergent gene pairs (*D*).

To determine whether the increase in γH2A and Rad52 foci after loss of the checkpoint inhibition of origin firing (Fig. 2) is due to genome instability at specific loci, we decided to map the location of these DNA damage markers. γ-H2A ChIP shows that in the wild-type strain, γ-H2A accumulated around early origins but not late origins (Fig. 3A; Supplemental Fig. S3A). This likely reflects the fact that γ-H2A only accumulates at replicating loci. In accordance with this, the *sld3-A dbf4-A* strain, which allows initiation at early and late origins in HU (Fig. 1A), accumulated γ-H2A around both early and late firing origins (Fig. 3A; Supplemental Fig. S3A). This γ-H2A ChIP was specific for the modified form of H2A, as no enrichment was observed in strains containing the *h2a-S129A* mutation, which lacks the phosphorylated serine (red lines, Fig. 3A), nor in strains lacking the kinases that phosphorylate H2A-S129 (*mec1Δ tel1Δ*, blue lines, Fig. 3A).

To determine whether γ-H2A preferentially accumulated at specific loci, we analyzed the location of the γ-H2A peaks. Showing chromosome XI as a representative snapshot of the genome, the peaks of γ-H2A were distributed throughout the chromosome for both the wild-type and *sld3-A dbf4-A* strains (Fig. 3B). For *sld3-A dbf4-A*, there are some unique peaks compared to wild type at normally late firing origins (e.g., orange arrow, Fig. 3B), consistent with *sld3-A dbf4-A* permitting replication initiation at those origins (see Supplemental Fig. S3B for overlay between γ-H2A and replication). Despite this, there are some sites that replicate efficiently in the *sld3-A dbf4-A* strain but accumulate only a small amount of γ-H2A (such as ARS1107, black arrow, Fig. 3B), and conversely there are other origins where a small increase in replication leads to a greater γ-H2A signal (e.g., ARS1123, pink arrow, Fig. 3B). Such differences are suggestive of some genomic bias in γ-H2A accumulation.

To identify loci that are susceptible to damage and to account for differences in replication between the strains, we normalized the γ-H2A signal at each genomic locus to the amount of replication at that location. From this analysis, we did not observe a correlation between γ-H2A and tRNA genes, telomeres, Ty elements, LTRs, and centromeres (data not shown). We did however observe a significant enrichment of γ-H2A in the *sld3-A dbf4-A* strain at gene pairs where the direction of transcription converges upon the direction of replication (hereafter called convergent gene pairs) (Fig. 3C). This correlation was specific to convergent gene pairs, not codirectional or divergent gene pairs (Fig. 3D). Interestingly, the enrichment of γ-H2A at convergent gene pairs increased with T_rep_ (Fig. 3C). This correlation is not due to a bias in the distribution of convergent gene pairs around late origins (Supplemental Fig. S3C).

To confirm the γ-H2A DNA damage mapping results, we also performed ChIP for Rad52-GFP from cells treated with MMS for 90 and 180 min (Supplemental Fig. S4). This anti-GFP ChIP showed great similarity with the γ-H2A ChIP, in that Rad52 was distributed throughout the genome, with enrichment at convergent gene pairs, not at nonconvergent gene pairs (Supplemental Fig. S4). Together, these ChIP analyses show that in the absence of checkpoint inhibition of origin firing, DNA damage markers appear throughout the genome, with some enrichment at convergently transcribed gene pairs.

### Genetic screens identify pathways that are important in the absence of checkpoint inhibition of origin firing

The mapping of DNA damage markers (Fig. 3) suggested that the failure to inhibit origin firing in the *sld3-A dbf4-A* strain causes DNA damage throughout the genome. To identify in an unbiased way the potential cause of such DNA damage, we conducted genetic screens between the *sld3-A dbf4-A* alleles and the entire yeast gene knockout collection (Addinall et al. 2011; Holstein et al. 2018). Many essential genes were also represented in this screen by including the DAmP (Decreased Abundance by mRNA Perturbation) allele collection, whereby mRNAs are destabilized through perturbation of the 3′ UTR (Breslow et al. 2008). For this screen, we used a quantitative fitness analysis (QFA) approach, which is a high-throughput growth analysis method in solid medium (Addinall et al. 2011; Holstein et al. 2018).

**Figure 4.**
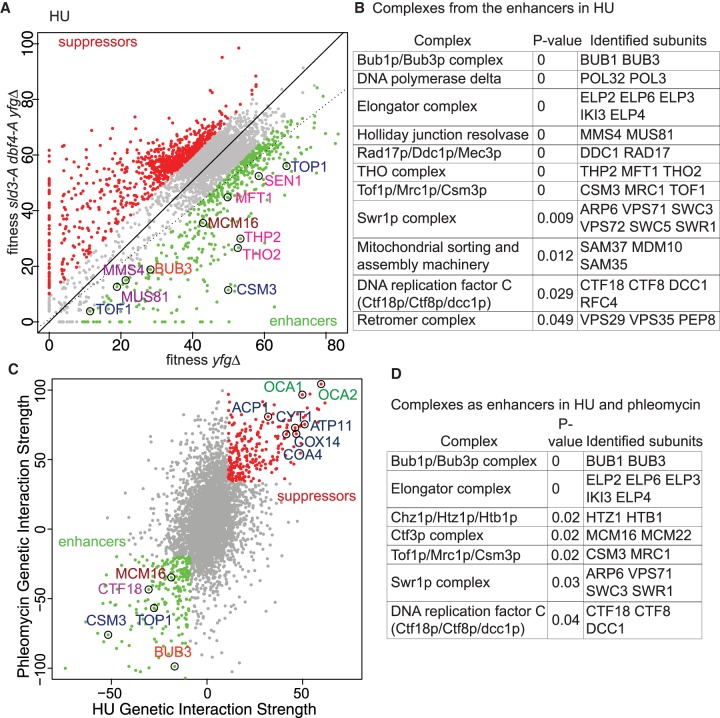
Genetic screens identify pathways that are important in the absence of checkpoint inhibition of origin firing. (*A*) Scatter plot of the fitness of the yeast genome knockout collection grown in 100 mM HU with (*y*-axis) or without (*x*-axis) the *sld3-A dbf4-A* alleles. Each dot corresponds to a different gene deletion. The top 25% of gene deletions (yfg = your favorite gene) that significantly enhance (green) or suppress (red) the fitness of *sld3-A dbf4-A* are indicated. The line of hypothetical equal fitness (dotted line) and the line of equal growth derived from a population model of the actual fitness of all the strains (solid line) are indicated. Several examples of the enhancer hits are highlighted and color-coded according to complex/function. (*B*) Analysis of the enriched protein complexes of the enhancers in *A*. (*C*) Genetic interaction strength (GIS) comparison between the screen in *A* and an equivalent screen performed in 0.5 µg/mL phleomycin. Enhancers and suppressors are highlighted as in *A*. Several examples of the enhancer/suppressor hits are highlighted and color-coded according to complex/function. The suppressors in blue are involved in mitochondrial function. (*D*) Analysis of the enriched protein complexes of the enhancers in *C*.

The fitness of every gene deletion (your favorite gene deletion, *yfgΔ*) and *yfgΔ sld3-A dbf4-A* was measured in quadruplicate in HU (Fig. 4A; Supplemental Table S1). Figure 4A highlights the *yfgΔ sld3-A dbf4-A* strains that grew significantly better than the equivalent *yfgΔ* alone (red dots, Fig. 4A), and these *yfgΔ* are hereafter classified as *sld3-A dbf4-A* suppressors. Conversely, the *yfgΔ* mutations that conferred worse growth with *sld3-A dbf4-A* are classified as enhancers (green dots, Fig. 4A). Gene ontology (GO) analysis of the enhancers in HU revealed an enrichment for genes involved in DNA/RNA metabolism and chromosome fidelity (Supplemental Fig. S5A; Supplemental Table S2). We validated this screen by generating null alleles of 17 of the enhancer hits in a different yeast strain (W303) and confirming the growth defect with *sld3-A dbf4-A* (data not shown). Analysis of the *sld3-A dbf4-A* enhancers relative to known protein complexes (Pu et al. 2009) identified several complexes as significant hits (Fig. 4A,B), including the THO complex, which is required for the resolution of R-loops, the Holliday junction resolvase Mus81/Mms4, and the CTM (Csm3/Tof1/Mrc1) complex, which maintains fork stability (Schalbetter et al. 2015; Brambati et al. 2018; Duch et al. 2018).

To further focus on the pathways that are important in the absence of checkpoint inhibition of origin firing, we also performed the genetic screen using another genotoxic agent, phleomycin. In order to compare directly between screens, a genetic interaction strength (GIS) score (Addinall et al. 2011) was used to define the relative growth of each *yfgΔ sld3-A dbf4-A* strain compared to the modeled average fitness of the population of strains (black line, Fig. 4A for HU). A negative GIS for a *yfgΔ*, indicates worse growth than expected when combined with *sld3-A dbf4-A* (enhancers), while a positive GIS indicates better growth of *yfgΔ sld3-A dbf4-A* than expected (suppressors). Plotting the GIS scores for the HU hits against the phleomycin hits highlights genes identified by both screens (Fig. 4C; Supplemental Table S3). The HU and phleomycin screens showed a high degree of overlap between the enhancers and suppressors (Supplemental Fig. S5B). As in Figure 4B, we identified protein complexes that were enriched as enhancers or suppressors in both screens (Fig. 4D; Supplemental Fig. S5C). Notable enhancers (Fig. 4C,D) include the type I topoisomerase Top1 and the CTM complex, as well as genes required for chromosome transmission fidelity, such as spindle assembly checkpoint (Bub1, Bub3), kinetochore (Mcm16, Mcm22), and cohesin loading factors (Ctf18 complex). The elongator complex was also identified in other QFA screens (Addinall et al. 2011), suggesting that it may be a false positive.

A similar analysis of the suppressors revealed genes required for mitochondrial function as well as the OCA tyrosine phosphatase complex (Fig. 4C; Supplemental Fig. S5C). Both mitochondrial mutants and the OCA complex were identified as suppressors of uncapped telomeres (Addinall et al. 2008), suggesting that they are common false positives or that they suppress multiple pathways of genome instability. Together, these genome-wide genetic screens identified key chromosomal maintenance pathways that are necessary for survival in the absence of checkpoint inhibition of origin firing during replication stress.

A longstanding hypothesis for the role of the inhibition of origin firing after DNA damage is to create a time window for repair to occur (Painter and Young 1980; Paulovich and Hartwell 1995). From this, we would expect mutations in repair pathways to be significant enhancer hits from these screens, but this was not the case (Supplemental Tables S1, S3). To further examine this hypothesis, we made mutations in eight different repair pathways and tested their genetic interactions with *sld3-A dbf4-A* in a range of different DNA damaging agents (Supplemental Fig. S5D). Consistent with the formation of Rad52 foci in the *sld3-A dbf4-A* strain, we did observe synthetic sickness between *sld3-A dbf4-A* and null mutations in *RAD52* and another HR factor *RAD50* but not with any other DNA repair mutation (Supplemental Fig. S5D). This suggests that facilitating repair of exogenous damage is not a major physiological role of the checkpoint inhibition of origin firing (see Discussion).

### Checkpoint inhibition of origin firing prevents excess catenation and chromosome loss

DNA replication generates supercoiling ahead of the fork, which is relieved by topoisomerases. This supercoiling can also be converted into catenanes behind the fork (precatenanes) by fork rotation, which is likely to be particularly important when topoisomerase action is restricted; for example, during replication termination (Peter et al. 1998; Schalbetter et al. 2015). The unbiased genetic screens showed that Top1, which removes supercoiling, and Csm3, which restrains fork rotation, are important for viability in the *sld3-A dbf4-A* strain (Fig. 4). From these genetic interactions, together with the dramatic increase in fork number in the *sld3-A dbf4-A* strain (Fig. 1), we hypothesized that there might be an increase in topological problems when the checkpoint fails to limit origin firing.

**Figure 5.**
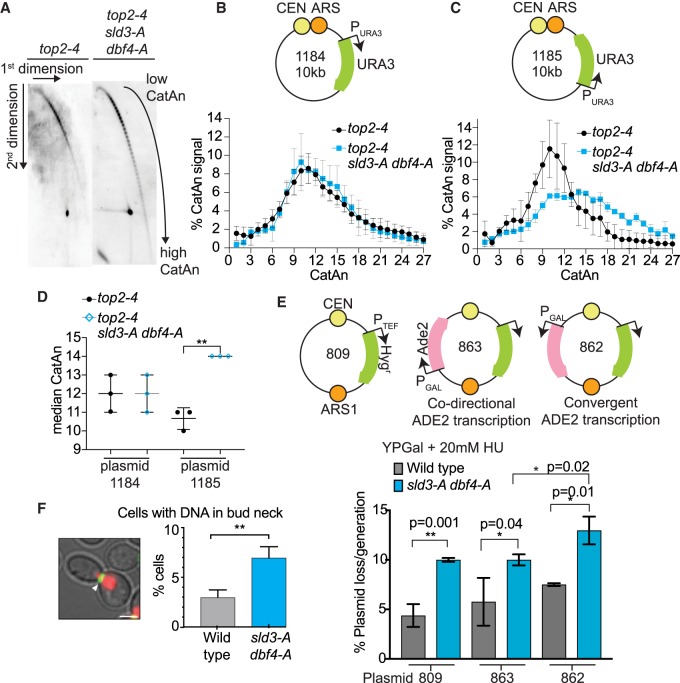
Checkpoint inhibition of origin firing prevents excess catenation and chromosome loss. (*A*) Southern blots of 2D gels from yeast containing the plasmid 1185 (see *C*). The indicated yeast strains were arrested in alpha factor at 25°C, then switched to the nonpermissive temperature (37°C) for *top2-4* and released into 0.033% MMS for 90 min. After nicking of DNA to remove supercoiling, the catenated forms (CatA) of the replicated plasmid can be discriminated. (*B*,*C, top*) Schematic diagram of plasmids with codirectional (1184) or convergent (1185) URA3 transcription relative to the direction of replication. (*Bottom*) Plot of the distribution of catenated isoforms of the plasmids 1184 (*B*) and 1185 (*C*) from the indicated strains. Error bars are SD, *n* = 3. (*D*) Graph of the median CatAn from *B* and *C*. Error bars are SD, *n* = 3. (*E*) Plasmid loss assay of the plasmids shown schematically above. Strains were grown overnight in YP galactose + 20 mM HU. Error bars are SD from *n* = 3. *P*-values are from paired *t*-tests. (*F*) Quantification of DNA in the bud neck after cytokinetic ring contraction. Error bars are SD from *n* = 3. Image of yeast (*left*) containing Htb2-mcherry (red) and myo1-GFP (green). Scale bar, 3 µm. A contracted myosin ring was considered to be <2 µm (white arrow).

To test this, we used an in vivo plasmid-based assay that detects the degree of supercoiling and fork rotation during replication through the accumulation of catenanes (CatA) (Schalbetter et al. 2015). This assay is performed in the absence of Top2 (here we use the conditional mutant *top2-4*) to ensure that catenanes are preserved after replication (Fig. 5A). Two-dimensional gel analysis of plasmids replicated in MMS showed that there is little difference between the wild-type and *sld3-A dbf4-A* strain in the catenation of a plasmid where the replication fork and the transcription unit are codirectional (plasmid 1184) (Fig. 5B,D). Interestingly, however, when we flipped the orientation of the marker gene so that transcription and replication are convergent on the plasmid, we observed an increase in the median number of catenanes specifically in the *sld3-A dbf4-A* strain (plasmid 1185, Fig. 5A,C,D). These data show that checkpoint inhibition of origin firing prevents the accumulation of topological problems during S-phase at sites of convergent transcription and replication.

Failure to remove catenanes results in nondisjunction in mitosis (Holm et al. 1989), and we wondered whether the excessive topological constraints resulting from failure of the checkpoint to inhibit origin firing might also result in chromosomal abnormalities. Failure to inhibit origin firing indeed resulted in a twofold increased loss-rate of a plasmid in HU (plasmid 809, Fig. 5E), indicative of increased chromosome loss. This plasmid-loss phenotype was not due to differences in origin firing, as this plasmid initiates replication early and fires equally in both the wild-type and *sld3-A dbf4-A* strains, as expected (Supplemental Fig. S6A).

As we observed increased topological problems due to convergence between replication and transcription (Fig. 5A–D), we wondered whether such conflicts might render a plasmid more susceptible to loss in the *sld3-A dbf4-A* strain. To test this, we added an additional ADE2 marker to the plasmid, transcribed either codirectionally with replication (plasmid 863) or transcribed convergently to the replication fork (plasmid 862, Fig. 5E). Although the codirectional plasmid was lost as frequently as the parental plasmid 809, convergent transcription indeed resulted in greater plasmid loss in the *sld3-A dbf4-A* strain in HU (Fig. 5E). The enhanced plasmid loss due to convergent transcription was unlikely due to inhibition of the plasmid origin because we did not observe this effect in the absence of HU (Supplemental Fig. S6B).

To further detect chromosomal abnormalities, we analyzed the transmission of yeast chromosomes during mitosis. Using myosin-GFP to label the contractile ring during cytokinesis and histone H2B-mCherry to visualize chromosomes, we measured the persistence of mitotic chromosomes in the bud neck, which is indicative of incomplete replication and failed segregation (Amaral et al. 2016). Significantly, we observed an increase in chromosomal DNA persisting in the bud neck during ring contraction in the *sld3-A dbf4-A* strain (Fig. 5F), suggestive of delayed replication termination or decatenation in the absence of checkpoint inhibition of origin firing. Together these analyses show that under replication stress, the failure to inhibit origin firing causes topological problems to accumulate in S-phase (Fig. 5A–D) and results in defects in chromosome segregation (Fig. 5E,F), both of which are exacerbated at sites of convergent transcription-replication (Fig. 5B–E).

### Topological defects exacerbate the genetic interactions and underlie the accumulation of DNA damage in the *sld3-A dbf4-A* strain

Many of the pathways identified in the genetic screens (Fig. 4) are either required to resolve DNA topological problems (Top1 and Csm3/Tof1) or are required to suppress the genome instability that arises from topological problems such as R-loops (e.g., THO complex) (Tuduri et al. 2009; El Hage et al. 2010) or terminal replication structures (Mus81/Mms4) (Regairaz et al. 2011). Therefore, we hypothesized that if the topological issues caused by increased origin firing (Fig. 5) are physiologically important, then reducing topoisomerase activity together with *sld3-A dbf4-A* should enhance the genetic interactions with other chromosome maintenance pathways.

**Figure 6.**
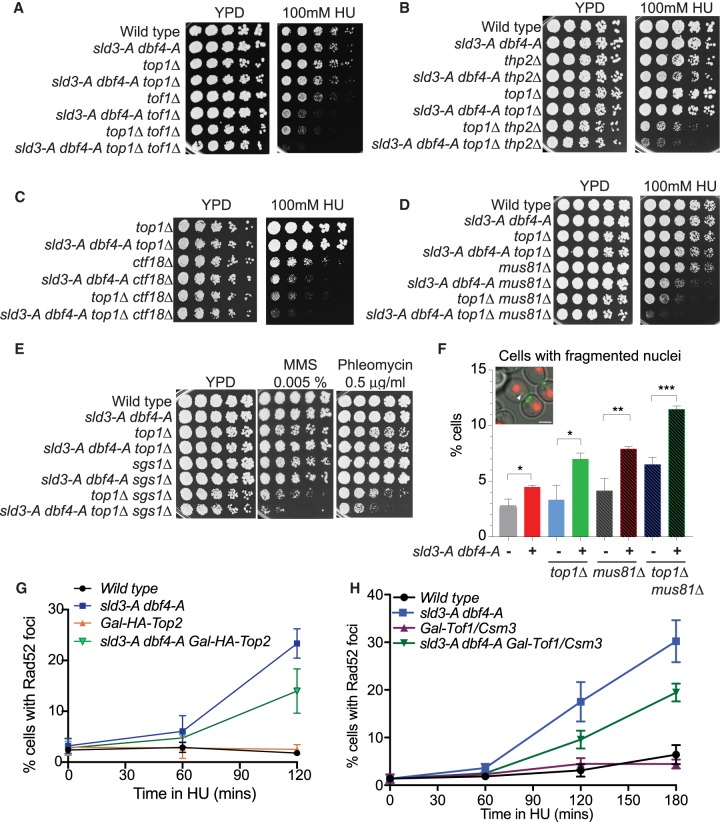
Topological defects explain the genetic interactions and DNA damage in the *sld3-A dbf4-A* strain. (*A–E*) Fivefold dilution growth assays of the indicated strains. (*F*) Quantification of nuclear fragmentation after nuclear separation in the indicated strains. Image of yeast containing Htb2-mcherry (red) and myo1-GFP (green) is shown. Fragmentation was considered only for Htb2 signal that was >0.1 µm from the rest of the nucleus (white arrow). Error bars are SD from *n* = 3. (*G*,*H*) Quantification of Rad52-GFP foci in the indicated strains released from G1 phase (0 min) into 200 mM HU in YP galactose medium. Error bars are SD, *n* = 3.

Combining *sld3-A dbf4-A* with a null mutation in the type I topoisomerase (*top1Δ*) indeed led to a much greater synthetic sickness with null mutations in genes required to prevent fork rotation (Tof1, Csm3) (Fig. 6A; Supplemental Fig. S6C). This genetic interaction was not specific to loss of Top1, as a hypomorphic mutation in Top2 also caused synthetic sickness with *sld3-A dbf4-A* and *tof1Δ* (Supplemental Fig. S6D). These genetic interactions suggest that the topological problems generated by global origin firing (Fig. 5A–D) become overwhelming when pathways that resolve supercoiling or catenation are compromised.

R-loops and chromosome nondisjunction are consequences of topological problems (Holm et al. 1989). Combining *sld3-A dbf4-A top1Δ* with a mutant defective in R-loop resolution (*thp2Δ*, Fig. 6B), as well as chromosome segregation mutants *bub3Δ* and *ctf18Δ* (Fig. 6C; Supplemental Fig. S6F), indeed led to a synergistic synthetic sickness. Relief of topological problems is also particularly important at replication termination, when converging forks meet (Branzei and Foiani 2010). As a result, we observed a robust synthetic sickness between *sld3-A dbf4-A top1Δ* and null mutations in the Mus81/Mms4 complex (Fig. 6D; Supplemental Fig. S6G) as well as the Sgs1 helicase (Fig. 6E), which are important for resolving persistent and terminal replication intermediates (Regairaz et al. 2011; Cejka et al. 2012). In line with these genetic data, null mutations in Top1 and Mus81 combined with *sld3-A dbf4-A* caused increased levels of nuclear fragmentation after mitosis (Fig. 6F). Such fragmentation is indicative of failures in replication completion/chromosome segregation in these mutants. Together these data show that defects in topoisomerases greatly enhance the genetic interactions of *sld3-A dbf4-A* identified in Figure 4, suggesting that topological problems are a major consequence of loss of checkpoint control of origin firing.

DNA damage markers, such as Rad52 foci, accumulate genome-wide in the *sld3-A dbf4-A* strain, with some enrichment at convergently transcribed gene pairs (Fig. 3; Supplemental Fig. S4). If these DNA damage markers occur in response to topological problems, then we would expect them to increase when decatenation/relaxation activities are compromised, and conversely, we might expect them to be suppressed if topoisomerases are overexpressed. Indeed, we observed an increase in Rad52 foci in the *sld3-A dbf4-A* strains that also lack Tof1 (Supplemental Fig. S7A) and Top1 (Supplemental Fig. S7B). Unfortunately, efforts to suppress this DNA damage by overexpression of either Top2 or Top1 were hampered by the fact that overexpression of either protein causes genome instability and death in yeast (Nitiss et al. 2001; Sen et al. 2016). Despite this, we found that an N-terminally tagged Top2 was highly unstable, allowing it to be temporarily overexpressed in S-phase (Supplemental Fig. S7C). Importantly, expression of this unstable form of Top2 was sufficient to partially suppress the appearance of Rad52 foci in the *sld3-A dbf4-A* strain (Fig. 6G). We also observed a partial suppression of Rad52 foci by overexpressing Csm3 and Tof1, which prevent precatenane formation (Fig. 6H; Supplemental Fig. S7D). Together, the enhanced synthetic lethality and chromosomal defects by combining *sld3-A dbf4-A* and topoisomerase mutants (Fig. 6A–F), together with the partial suppression of DNA damage by overexpression of Top2 or Csm3/Tof1 (Fig. 6G,H), suggest that the accumulation of topological problems is a significant consequence of loss of checkpoint inhibition of origin firing.

### High rates of replication initiation in a normal S-phase causes similar phenotypes to failure of checkpoint inhibition of origin firing

**Figure 7.**
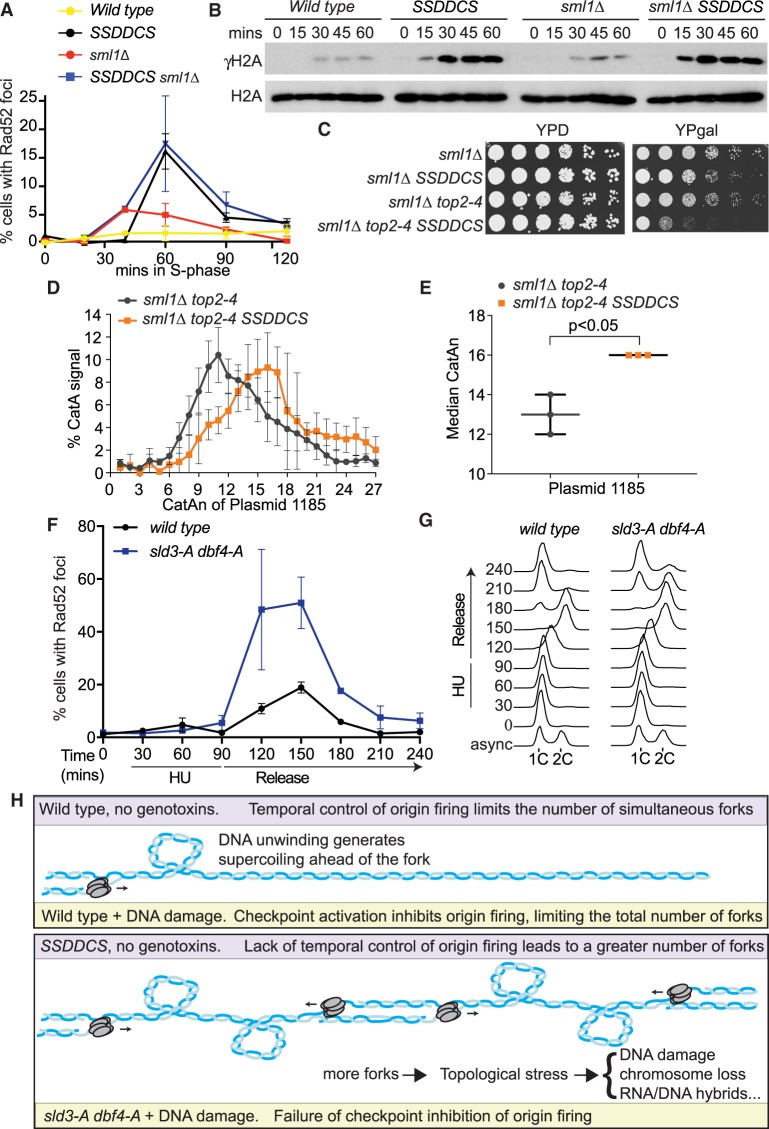
High rates of replication initiation in a normal S-phase causes similar phenotypes to failure of checkpoint inhibition of origin firing. (*A*) Quantification of Rad52-GFP foci in the indicated strains released from G1 phase arrest (0 min) into YP galactose medium. The SSDDCS strain expresses limiting replication factors from galactose-inducible promoters. Error bars are SD, *n* = 3. (*B*) Western blots of the indicated strains released from G1 phase arrest with alpha factor (0 min) into YP galactose medium. (*C*) Fivefold dilution growth assays of the indicated strains in the presence (YPgal) or absence (YPD) of expression of SSDDCS. (*D*) Plot of the distribution of catenated isoforms of the plasmid 1185 as in Figure 5C. Error bars are SD, *n* = 3. (*E*) Graph of the median CatAn from *D*. Error bars are SD, *n* = 3. (*F*,*G*) Quantification of Rad52-GFP foci (*F*) and flow cytometry (*G*) of strains released from G1 phase arrest with alpha factor (0 min) into 200 mM HU for 90 min and then washed into HU-free media (release) for a further 150 min. For *F*, error bars are SD, *n* = 3. (*H*) Model for the role of origin firing control in preventing topological stress. Wild-type cells (*top*) limit simultaneous fork number in a normal S-phase (purple) through a temporal order of origin firing and after DNA damage (yellow) through the checkpoint inhibition of origin firing. In the absence of the checkpoint inhibition of origin firing (*sld3-A dbf4-A*, yellow, *bottom*) or in the *SSDDCS* strain in a normal S-phase (purple, *bottom*), excess origin firing creates topological problems and increased reliance on pathways to remove supercoils and catenanes. Failure to deal with this stress leads to DNA damage/chromosome loss, possibly through increased RNA/DNA hybrid formation or fork reversal.

Thus far we have used the separation of function mutants, *sld3-A dbf4-A*, to show that increased replication initiation after replication stress leads to topological problems, subsequent DNA damage, and genome instability (Figs. 5, 6). It is unclear from these experiments what the importance of replication stress is in creating these problems. Perhaps replication stress generates an increased dependence on topoisomerase or fork rotation activities, possibly due to DNA repair. Alternatively, it may be that the excessive number of replication forks is sufficient to cause topological problems and subsequent DNA damage. To distinguish between these possibilities, we utilized a yeast strain that can conditionally increase the number of replication initiation events in a normal S-phase (Mantiero et al. 2011). Overexpression of limiting replication factors (Sld3, Sld2, Dpb11, Dbf4, Cdc45, and Sld7, abbreviated to SSDDCS) causes many origins to fire earlier than they would in a normal cell cycle, resulting in a faster S-phase (Mantiero et al. 2011). Importantly, we show that overexpression of limiting replication factors in a single cell cycle, in the complete absence of exogenous DNA damage or fork stalling agents, also leads to the accumulation of both Rad52 foci and γH2A in S-phase (Fig. 7A,B).

Increased rates of replication in a normal S-phase leads to the depletion of dNTPs and Rad53 activation, which can be suppressed by the deletion of the RNR inhibitor *SML1* (Mantiero et al. 2011). Deletion of *SML1* did not affect the accumulation of Rad52 foci, nor γH2A, suggesting that this is not a consequence of dNTP depletion or Rad53 activation (Fig. 7A,B). We also observed that the SSDDCS strain exhibits synthetic sickness with a hypomorphic mutant of Top2 (Fig. 7C), suggesting that excessive origin firing in a normal S-phase indeed leads to greater dependence on topoisomerases. Importantly, we observed that high rates of initiation in a normal S-phase also resulted in increased catenation of a replicated plasmid in vivo (Fig. 7D,E). These data show that the phenotypes associated with the *sld3-A dbf4-A* strain undergoing replication stress, such as DNA damage (Fig. 2), genetic interactions with topoisomerases (Figs. 4, 6), and accumulation of catenanes on a mini-chromosome in vivo (Fig. 5A–D), also occur when the levels of replication initiation are increased in an otherwise normal S-phase.

If it is the excess of normal forks, rather than stalled forks, that causes topological problems, then we hypothesized that DNA damage should occur in the *sld3-A dbf4-A* strain even after HU treatment, when fork stalling has abated. To test this, we first arrested yeast cells in 200 mM HU for 90 min to allow almost all origins to fire and stall in the *sld3-A dbf4-A* strain (Fig. 1A), and then we released the cells into fresh medium lacking HU to observe when Rad52 foci accumulate. After 90 min in HU, when most forks are stalled and Rad53 is active (Supplemental Fig. S7F), we observed very low amounts of Rad52 foci (Fig. 7F). This suggests that fork stalling by itself is not the cause of Rad52 foci in the *sld3-A dbf4-A* strain. Importantly when we released these cells in the absence of HU, allowing forks to progress and terminate, we observed a dramatic increase in Rad52 foci, coincident with S-phase progression (Fig. 7F,G). The *sld3-A dbf4-A* strain exhibited delayed progression through mitosis (Fig. 7G), consistent with defects in the completion of replication in this strain in HU (Fig. 5F). Together, these experiments suggest that excessive replication initiation, followed by high levels of normal fork progression, is an important driver of topological stress and the accumulation of DNA damage markers.

## Discussion

### Role of checkpoint inhibition of origin firing

Over 40 years ago, it was first established that eukaryotes inhibit replication initiation in the face of DNA damage (Painter 1977). By using specific separation of function mutants, our data suggest that the checkpoint limits the total number of simultaneous forks in order to maintain the balance between fork progression and topoisomerase/fork rotation activities (Fig. 7H). We provide direct evidence that, under conditions when excessive origins fire, both in the presence of DNA damage and in a normal S-phase, topological linkages accumulate in vivo (Figs. 5A–D, 7D,E). Failure to resolve supercoiling and catenation in a timely manner is a likely source for the DNA damage and chromosome segregation defects that occur when excessive origins fire, and this DNA damage can indeed be suppressed by overexpression of topoisomerase (Fig. 6). Since transcription also generates positive supercoiling, failure to resolve topological problems may explain why convergent replication-transcription units are prone to accumulate excess catenation (Fig. 5A–D) and why convergently transcribed gene pairs accumulate DNA damage in the *sld3-A dbf4-A* strain (Fig. 3C). The overwhelming of topoisomerase activities also explains why topological problems can occur in *trans* on episomal plasmids with only a single origin (Figs. 5A–D, 7D,E). As Top1 is known to bind to the replisome, possibly through interactions with Tof1 (Bell and Labib 2016), large numbers of replication forks progressing simultaneously might result in depletion of topoisomerase activities, affecting forks in *trans*. Consistent with our model (Fig. 7H), DNA damage accumulates during normal fork progression (when topoisomerases are required), not during fork stalling (Fig. 7A–G).

A longstanding hypothesis for the role of the checkpoint in delaying S-phase progression is that blocking origin firing allows more time for DNA repair to occur (Painter and Young 1980; Paulovich et al. 1997). Our data suggest that this is not a primary role for the checkpoint inhibition of firing. First of all, the whole-genome and targeted screens did not identify genetic interactions between *sld3-A dbf4-A* and many repair pathways (Supplemental Tables S1, S3; Supplemental Fig. S5D). Furthermore, a strain that fires multiple origins simultaneously, even in the absence of any exogenous genotoxins, also causes DNA damage and the accumulation of topological problems (Fig. 7A–E). In addition, fork stalling is not the driver of Rad52 foci accumulation in the *sld3-A dbf4-A* strain (Fig. 7F,G). Rather than needing time for repair, stalled forks are actually rescued by forks emanating from neighboring dormant origins even in checkpoint-proficient cells (McIntosh and Blow 2012). Our data suggest that it is the global level of origin firing which is important to prevent topological constraints during S-phase, irrespective of exogenous DNA damage (Fig. 7H).

While we show that topological defects are important to generate Rad52 foci (Fig. 6G,H), the function of Rad52 in the absence of checkpoint inhibition of origin firing is not clear. We cannot rule out that DSBs do form at some point due to excess origin firing, but we note that HR proteins resolve replication fork intermediates in the absence of DSBs (Kolinjivadi et al. 2017; Ait Saada et al. 2018). Indeed, a significant consequence of topological defects is fork reversal, whereby positive supercoiling ahead of the replisome drives nascent DNA at the fork to regress and anneal to generate a four-way junction (Postow et al. 2001; Neelsen and Lopes 2015). Interestingly, a recent study has shown a role for Rad52 in protection of reversed forks from degradation (Malacaria et al. 2019). It is also notable that Rad52 foci and γH2A occur in response to RNA-DNA hybrids (Costantino and Koshland 2018; García-Rubio et al. 2018). The genetic interactions with pathways required for R-loop resolution and the accumulation of DNA damage markers at convergent genes (Figs. 3C, 4A) suggests that R-loops may also be a consequence of excessive origin firing, possibly also as a downstream consequence of topological problems (Fig. 7H; Hamperl and Cimprich 2014).

Although we show here that topological stress is a prominent consequence of failure of checkpoint inhibition of origin firing, the synthetic lethality screens also identified other processes that may be affected by this checkpoint pathway (Fig. 4). One example is the histone variant H2A.Z (Htz1 in yeast) and its associated remodeling complex Swr1, which were significant hits from both screens (Fig. 4D). Swr1/Htz1 have roles in many cellular processes, such as transcription and chromatin maintenance (Morrison and Shen 2009). Despite this, we note that Swr1/Htz1 also have roles in response to replication stress and CTM complex function (Morrison and Shen 2009; Srivatsan et al. 2018), so their genetic interaction with *sld3-A dbf4-A* may still be related to the topological problems described here.

### Topological stress as a branch of replication stress

“Replication stress” is a frequently used term that encompasses a wide range of different genome maintenance events (Kotsantis et al. 2018). By utilizing separation of function mutants, here we have isolated a specific form of replication stress caused by too much origin firing (Fig. 7H). The identification of topological problems as a branch of replication stress may contribute to our understanding of the locations and mechanisms of genome instability. For example, sites of convergent replication-transcription conflicts have increased genome instability (Hamperl et al. 2017). The topological stress described here may underlie DNA damage at locations or under conditions that affect replication efficiency, transcriptional direction, supercoiling, and topoisomerase availability/accessibility.

Eukaryotes license many more origins than are necessary to ensure complete genome replication (McIntosh and Blow 2012). Our data show that topological stress is generated even in a normal S-phase when too many origins fire (Fig. 7C–E), perhaps explaining why most cells use only a subset of their potential origins. In the early embryonic divisions of many metazoa, such as flies and frogs, S-phase is incredibly short due to very high rates of replication initiation. These early divisions occur in the near absence of transcription, which may be one explanation for how these cells avoid genome instability, but it will be interesting to understand how embryonic cells, but not somatic cells, cope with high rates of topological stress. Furthermore, topoisomerase and checkpoint inhibitors are potential combinatorial therapies for the treatment of cancers (Josse et al. 2014; Thomas et al. 2018). This study reveals that unbridled origin firing creates an enhanced dependence on topoisomerase activity, which may provide a new mechanistic rationale for the use of combined checkpoint/topoisomerase inhibition therapies.

## Materials and methods

### Strains and growth conditions

All yeast strains are derived from W303-1a; see Supplemental Table S4. Cell growth, arrests, flow cytometry, and yeast protein extracts were as previously described (Zegerman and Diffley 2010).

### Replication profiles

Yeast genomic DNA was extracted using the spheroplast method (http://fangman-brewergeneticswashingtonedu/indexhtml). Samples were prepared according to the TruSeq Nano sample preparation guide from Illumina. To generate replication timing profiles, the ratio of uniquely mapped reads in the replicating samples to the nonreplicating samples was calculated following Müller et al. (2014), and profiles were smoothed by a Fourier transformation (Müller et al. 2014). A replication peak was defined as a curve point where the S to G1 ratio/Δkb changed from plus to minus and the same sign was kept at more than 3 kb from the change point. A peak is therefore defined as a local maximum. The values of T_rep_ were from OriDB (Siow et al. 2012).

### Chromatin immunoprecipitation-sequencing (ChIP-seq)

ChIP-seq was performed as previously described (Can et al. 2019). Antibodies for IP were anti-H2A (39945, Actif motif), IgG (AB27478, Abcam), or anti- γH2A (AB15083, Abcam) or anti-GFP (3h9, Chromotek).

### Whole-genome synthetic lethality screen

Synthetic Genetic Array (SGA) was used as described in Tong and Boone (2006) and Holstein et al. (2018) to create two independent strain libraries using a BM3 colony pinning robot (S&P Robotics). The starter strains used were DLY8000 *MAT alpha lyp1::HPHMH::NATMX can1delta::STE2pr-Sp_his5 his3Δ leu2Δ ura3Δ met15Δ p* LEU2-sld3-A dbf4-A and DLY7388 *MAT alpha lyp1::LEU2::HPHMH::NATMX can1delta::STE2pr-Sp_his5 his3Δ leu2Δ ura3Δ met15Δ* as control. The strain libraries used were the yeast knockout (YKO) collection (a kind gift from Charlie Boone) and the DAmP collection (purchased from Open Biosystems, now Dharmacon Horizon, catalog# YSC5090). Following SGA, we used Quantitative Fitness Analysis (QFA) (Addinall et al. 2008, 2011; Holstein et al. 2018) to determine the fitness of the strains within the two libraries. Independent 200-µL liquid cultures of each strain were grown to saturation using a BM3 colony pinning robot (S&P Robotics), diluted in sterile water, and spotted onto the same solid media used in the final SGA selection stage. This media was synthetic defined media (Formedium YNBMSG02) lacking the amino acids arginine, histidine, leucine, and lysine, with canavanine (50 µg/mL, Sigma C9758) and thialysine (50 µg/mL, Sigma A2636) and also containing the antibiotics G418 (200 µg/mL, Sigma A1720), ClonNat (100 µg/mL, Werner BioAgents 5.001.000), and hygromycin (300 µg/mL, Sigma H3274) (Holstein et al. 2018). The strains were spotted on the synthetic media which contained no compounds, 2 µg/mL phleomycin, or 50 mM hydroxyurea using a Beckman Coulter FX robot and photographed every 4 h over 5 d. Solid agar plates were photographed on a spImager (S&P Robotics) with an integrated camera. Manual settings of the camera were as follows: 0.25 sec; aperture, F10; white balance, 3700 K; ISO100; image size, large; image quality, fine; image type, .jpg. Culture density was generated from captured photographs using the integrated optical density measure of cell density provided by the image analysis tool Colonyzer. In order to calculate the fitness, the maximum doubling potential (MDP, population doublings) was multiplied by the maximum doubling rate (MDR, population doublings/day), and the mean value of four replicates was calculated (Tong and Boone 2006; Addinall et al. 2008, 2011; Holstein et al. 2018). The database used for the identification of enriched protein complexes was cyc2008 (Pu et al. 2009).

### Microscopy

Samples were plated onto 35-mm glass bottom plates (MatTek) precoated with Concanavalin A (Sigma). After 5 min, cells were imaged on a Deltavision widefield fluorescent microscope (GE Healthcare) using an Olympus 60× objective. Images were acquired, deconvoluted, and projected using SoftWoRx (GE Healthcare). Analysis of DNA in the bud neck utilized a plugin for FIJI. At least 200 cells were counted for every time point.

### Western blot

Detection of Rad53 was performed using ab104232 (Abcam, 1:5000), H2A with ab13923 (Abcam, 1:1000), and γH2A (phospho S129) with ab15083 (Abcam, 1:1000).

### Plasmid loss

Cultures were pregrown in selective medium (YPD + hygromycin, 500 µg/mL) and then diluted into nonselective medium YPGal + 20 mM HU, and grown overnight at 30°C. Once the cultures had reached mid-log phase, 100 cells were plate on YPD with or without hygromycin. The rate of plasmid loss per generation was calculated using the formula 100 × (1−RMP^1/G^), where RMP is the ratio of plasmid maintenance (number of colonies on YPD + hygromycin/number of colonies on YPD) and G is the number of generations during the overnight culture.

### Detection of plasmid catenation

Detection of plasmid catenation was performed as previously described (Schalbetter et al. 2015).

## Supplementary Material

Supplemental Material
