# Supplementary material for: Checkpoint inhibition of origin firing prevents DNA topological stress
Source: Genes Dev. 2019 Nov 1;33(21-22):1539–54. doi: 10.1101/gad.328682.119 (PMC6824463; doi:10.1101/gad.328682.119)
Supplement: Supplemental Material [file supp_33_21-22_1539__index.html]

Checkpoint inhibition of origin firing prevents DNA topological stress — Supplemental Material 

# Checkpoint inhibition of origin firing prevents DNA topological stress

## Supplemental Material

- Supplemental\_SuppTable2.xlsx
- SUPPLEMENTAL\_328682\_Fig7.ai
- Supplemental\_SuppTable3.xlsx
- Supplemental\_Figure\_Legends.pdf
- Supplemental\_SuppTable4.docx
- Supplemental\_SuppTable1.xlsx
- SUPPLEMENTAL\_328682\_Fig1.ai
- SUPPLEMENTAL\_328682\_Fig4.ai
- SUPPLEMENTAL\_328682\_Fig2.ai
- SUPPLEMENTAL\_328682\_Fig5.ai
- SUPPLEMENTAL\_328682\_Fig3.ai
- SUPPLEMENTAL\_328682\_Fig6.ai
