# Supplementary figures and images for: Checkpoint inhibition of origin firing prevents DNA topological stress
Source: Genes Dev. 2019 Nov 1;33(21-22):1539–54. doi: 10.1101/gad.328682.119 (PMC6824463; doi:10.1101/gad.328682.119)

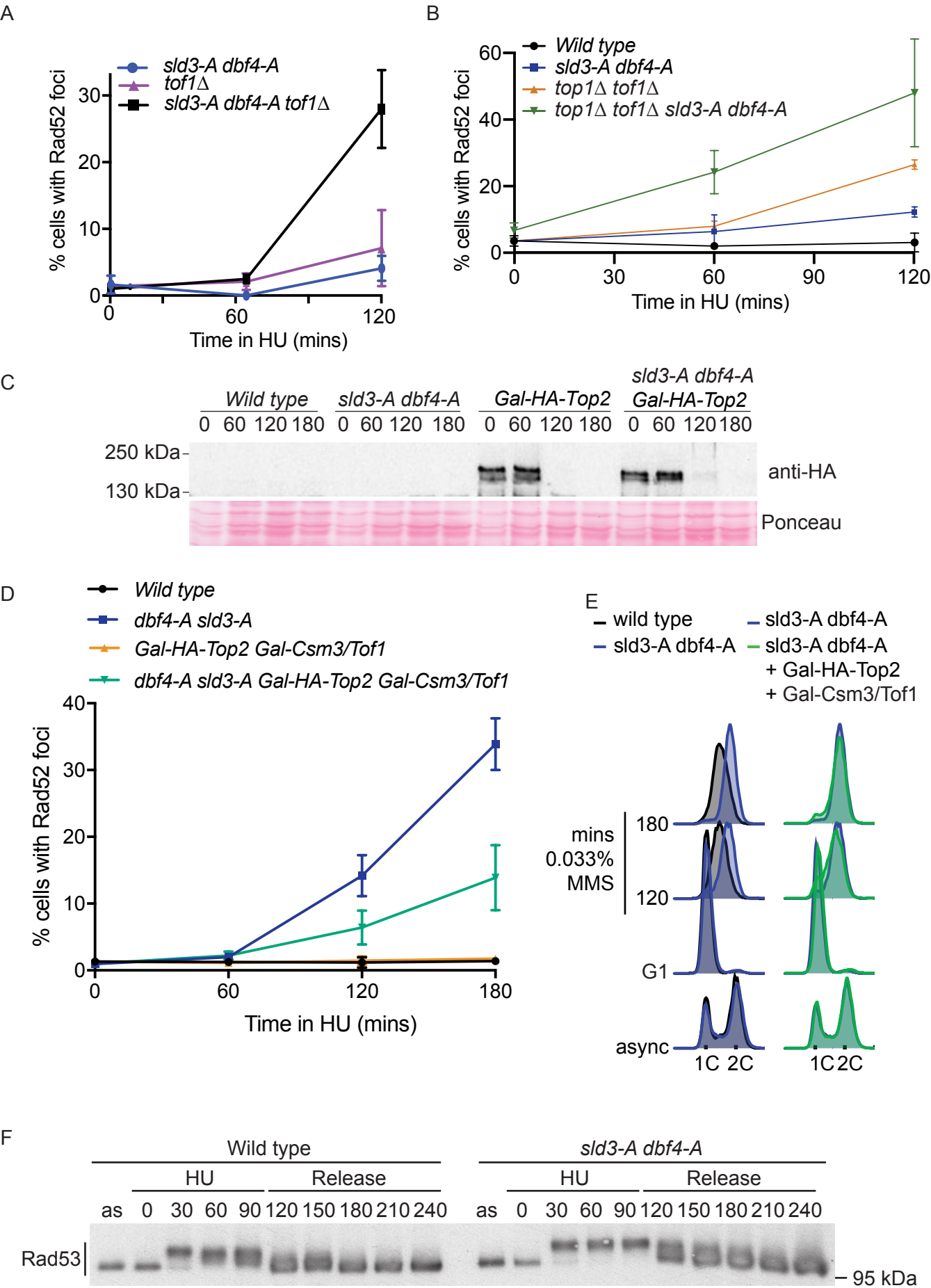

Supplement: Supplemental Material [file supp_gad.328682.119_SUPPLEMENTAL_328682_Fig7.ps]

Supplemental figure 1. Morafraille et al. 328682

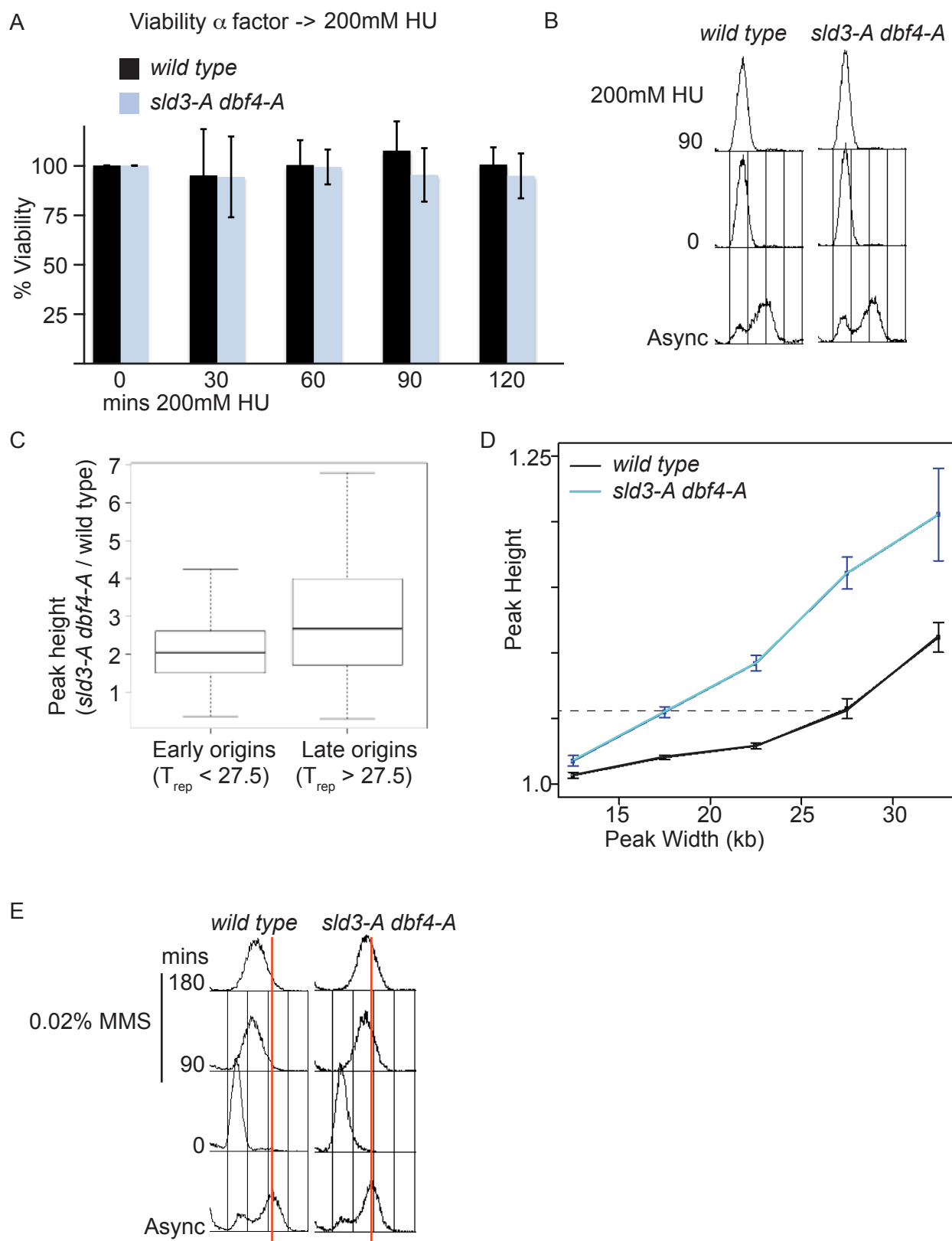

Supplement: Supplemental Material [file supp_gad.328682.119_SUPPLEMENTAL_328682_Fig1.ps]

Supplemental Figure 4. Morafraille et al. 328682

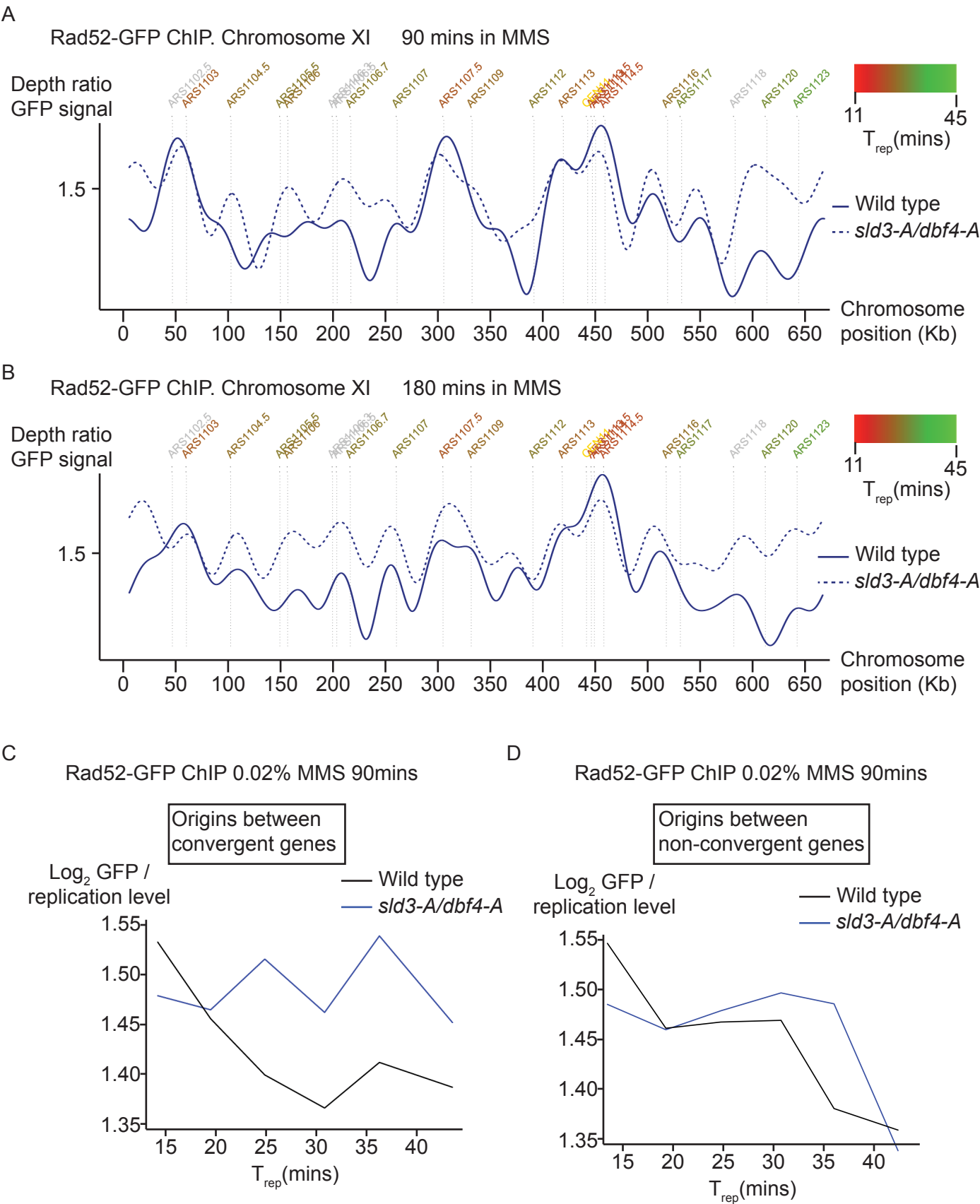

Supplement: Supplemental Material [file supp_gad.328682.119_SUPPLEMENTAL_328682_Fig4.ps]

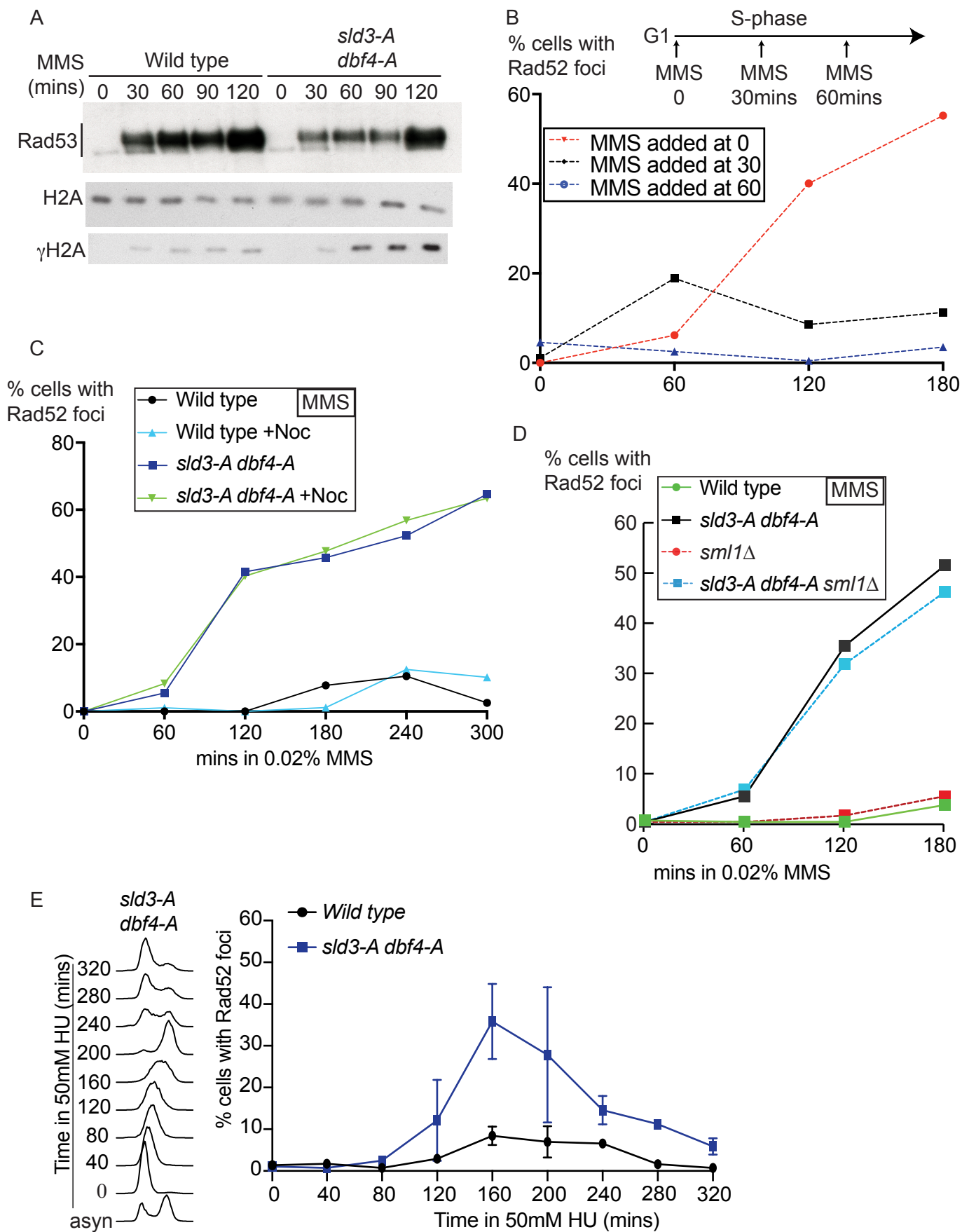

Supplement: Supplemental Material [file supp_gad.328682.119_SUPPLEMENTAL_328682_Fig2.ps]

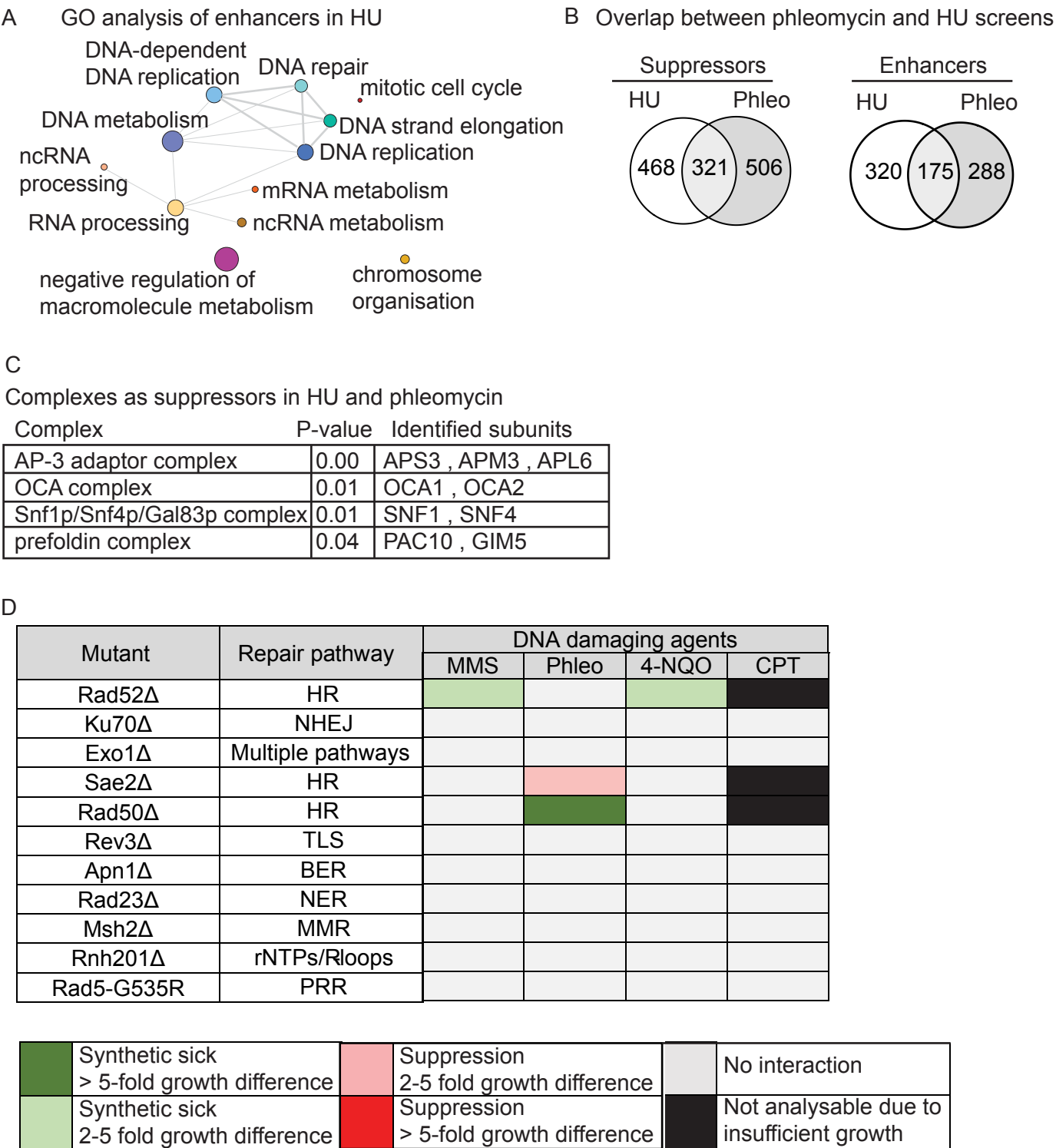

Supplement: Supplemental Material [file supp_gad.328682.119_SUPPLEMENTAL_328682_Fig5.ps]

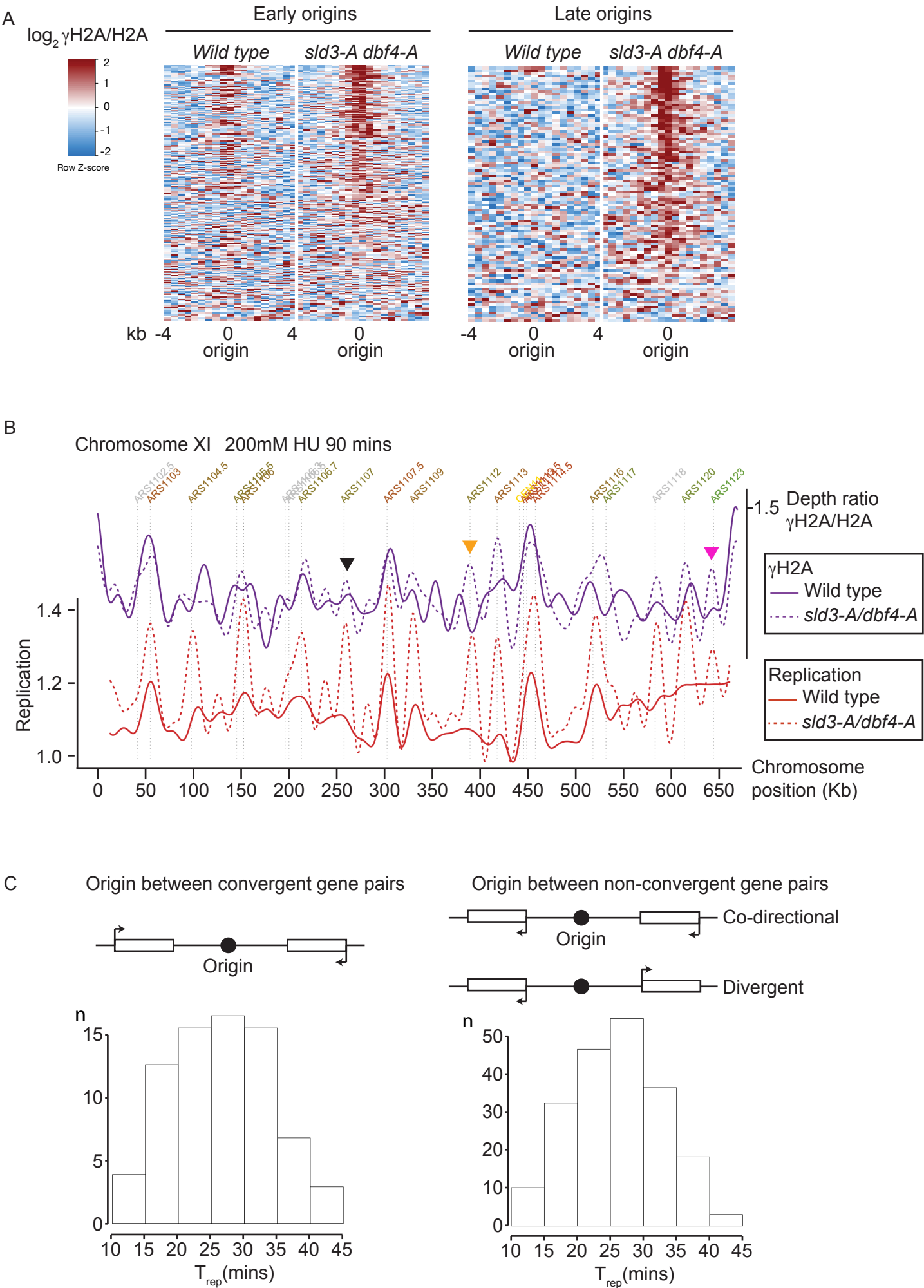

Supplement: Supplemental Material [file supp_gad.328682.119_SUPPLEMENTAL_328682_Fig3.ps]

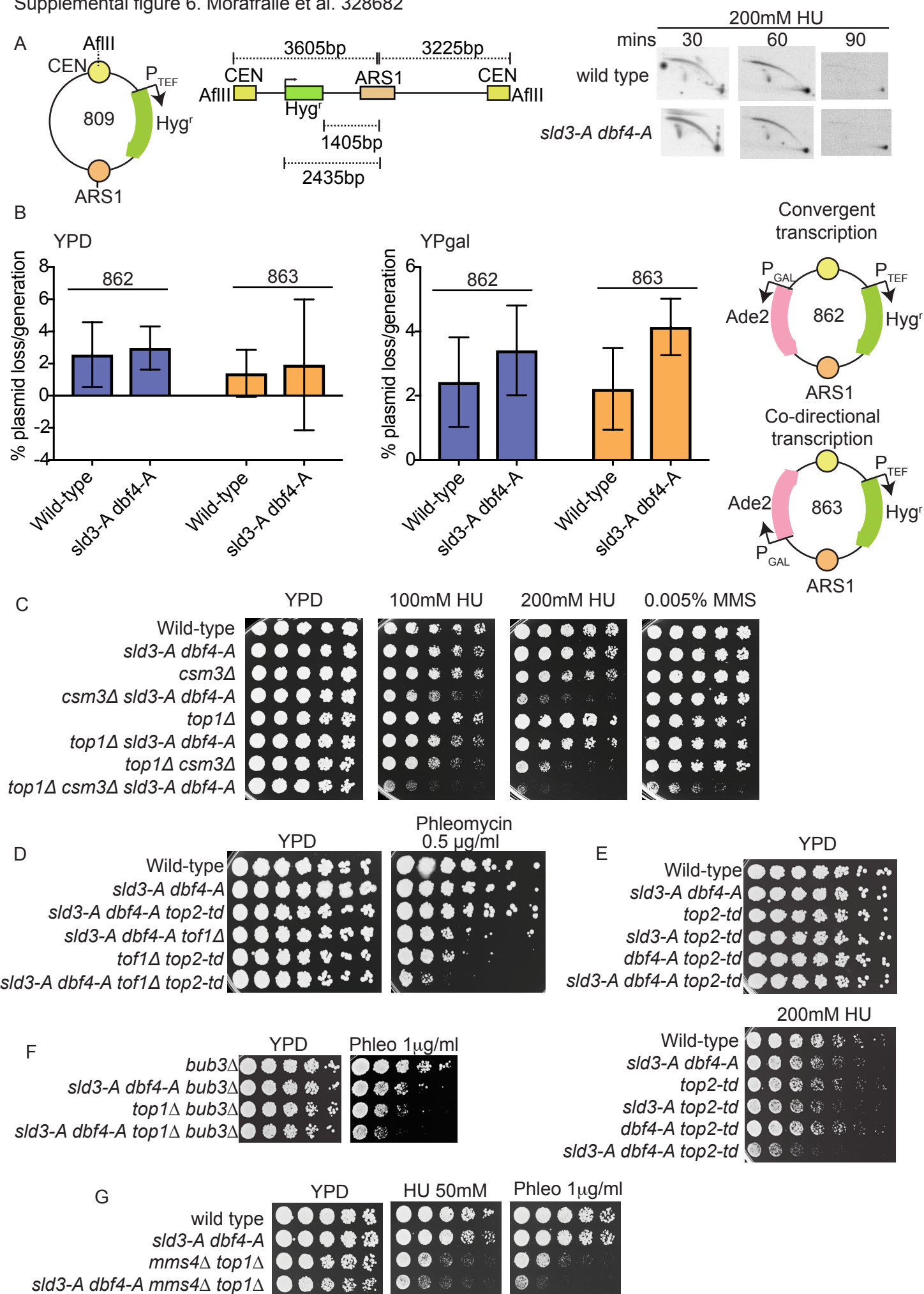

Supplement: Supplemental Material [file supp_gad.328682.119_SUPPLEMENTAL_328682_Fig6.ps]
