# Supplementary material for: Checkpoint inhibition of origin firing prevents DNA topological stress
Source: Genes Dev. 2019 Nov 1;33(21-22):1539–54. doi: 10.1101/gad.328682.119 (PMC6824463; doi:10.1101/gad.328682.119)
Supplement: Supplemental Material [file supp_gad.328682.119_Supplemental_Figure_Legends.pdf]

Supplemental Figure Legends

Supplemental Figure 1. *sld3-A dbf4-A* are effective separation of function mutants and prevent the checkpoint inhibition of origin firing globally.

A. Viability analysis of strains arrested in G1 phase arrest with alpha factor (0 mins) and then released into 200mM HU for the indicated times. For each time point 200 cells were plated onto YPD plates in duplicate to count colony growth (viability) after 2 days. The number of colonies at time 0 was set to 100%. Errors bars are SD, n=4. This shows that the *sld3-A dbf4-A* strain has wild type viability in HU, consistent with these alleles having normal replication functions and that other aspects of checkpoint function, such as fork stabilisation are not affected in this strain.

B. Flow cytometry of the experiment in Figure 1A.

C. Box plot of the peak heights of the *sld3-A dbf4-A* strain, divided by the wild type from Figure 1A. Data was binned into those origins that normally fire early or late (less than or more than the median T_{rep} value of 27.5 mins). Horizontal lines are median values, error bars are SD.

D. The peak widths from Figure 1A was binned by kb (x-axis) and was plotted against average peak height for that bin (y-axis). This graph shows that for a given peak height (see dotted line for example) peaks are wider in the wild type strain than in the *sld3-A dbf4-A* strain. Therefore although there are many more forks in the *sld3-A dbf4-A* strain, forks on average move less far in the *sld3-A dbf4-A* strain than in the wild type. Error bars are SD.

E. Flow cytometry of the experiment in Figure 1E/F. The *sld3-A dbf4-A* strain allows for a faster S-phase in the presence of MMS.

Supplemental Figure 2. Failure to inhibit origin firing in HU/MMS results in the accumulation of DNA damage markers.

A. Western blots from the indicated strains released from G1 phase arrest with alpha factor (0 mins) into 0.02% MMS for the indicated time points.

B. Quantification of Rad52-GFP foci in the *sld3-A dbf4-A* strain released from G1 phase arrest with alpha factor (0 mins). 0.02% MMS was either added to the culture immediately after release from G1 (0) or in mid to late S-phase (30 or 60 mins). Rad52-GFP foci accumulate in the *sld3-A dbf4-A* strain when MMS is added before S-phase begins (0).

C. As B. Addition of nocodazole to inhibit anaphase does not suppress the appearance of Rad52 foci in the *sld3-A dbf4-A* strain.

D. As B. Increasing nucleotide pools, through deletion of the RNR inhibitor *SML1*, does not suppress the appearance of Rad52 foci in the *sld3-A dbf4-A* strain.

E. As B, except strains were released into 50mM HU. The flow cytometry of the *sld3-A dbf4-A* strain from this experiment is shown on the left. Error bars are SD, n=3.

Supplemental Figure 3. Checkpoint inhibition of origin firing prevents DNA damage globally, in particular at convergently transcribed genes

A. The γH2A ChIP data from Figure 3A represented as a heatmap, centred on the origin, with 4kb either side. Early origins have a T_{rep} value < 27.5 mins, late origins have a T_{rep} value > 27.5 mins.

B. Overlay of the replication data from Figure 1A (red) with the γH2A ChIP data Figure 3B (purple). This shows sites that are highly replicated in the *sld3-A dbf4-A* strain, but accumulate little γH2A (e.g black arrow) or vice versa (e.g pink arrow).

C. (Top) Schematic diagram of an origin situated between a convergently transcribed pair of genes (left) or a non-convergently transcribed pair of genes (right). Non-convergent transcripts can be either co-directional with respect to each other or divergent. (Below) Histogram of the gene pairs surrounding all origins binned according their median time of replication in a normal S-phase (T_{rep}). Convergent or non-convergent gene pairs show no differences in the T_{rep} of the neighbouring origin.

Supplemental Figure 4. Rad52-GFP ChIP confirms that checkpoint inhibition of origin firing prevents DNA damage globally, in particular at convergently transcribed gene pairs.

A and **B.** Anti-GFP ChIP of Rad52-GFP from the indicated strains, after release from alpha factor into 0.02% MMS for 90 (A) or 180 minutes (B). Only chromosome XI is shown for simplicity.

C and **D**. The GFP ChIP signal at origins from A was binned according to their average time of replication in a normal S-phase (T_{rep}). The data is split between those loci that contain convergent gene pairs (C) or non-convergent gene pairs (D). The y-axis is the GFP signal normalised to the amount of replication at that genomic locus.

Supplemental Figure 5. Genetic screens identify pathways that are important in the absence of checkpoint-inhibition of origin firing.

A. GO analysis of the enhancer hits from the fitness screen in HU with the yeast genome knock out collection from Figure 4A.

B. Venn diagram of the overlap of the statistically significant hits from the HU and Phleomycin whole genome genetic screens.

C. Analysis of the enriched protein complexes from the suppressors in Figure 4C.

D. Targeted genetic analysis of repair pathway mutations in combination with *sld3-A dbf4-A* in yeast strain W303. Data from 5-fold growth dilution assays as in Figure 6A-E are summarised here as a table, colour coded according to whether any genetic interaction was observed between *gene*Δ and the *gene*Δ *sld3-A dbf4-A* triple mutant. All strains apart from *rad5-G535R* are *RAD5*$^{+}$.

Supplemental Figure 6. Plasmid loss is not affected in the *sld3-A dbf4-A* strain in the absence of replication stress and topological defects explains the genetic interactions in the sld3-A dbf4-A strain

A. 2D gel analysis of the plasmid 809 (left) digested with AflII from the indicated strains released from alpha factor into 200mM HU. A scale diagram of the plasmid after linearization with AflII is shown (middle). As expected, episomal plasmids replicate early and therefore fire equally in the wild type and the *sld3-A dbf4-A* strain.

B. Plasmid loss analysis of the indicated plasmids in the absence of genotoxic stress. In the absence of checkpoint activation, a normal temporal programme of origin firing occurs and the wild type and *sld3-A dbf4-A* strains therefore have the same plasmid loss rates. Comparison between YPD and YP + galactose, which induces transcription from the Ade2 gene on the plasmid, shows no effect of transcription specifically on plasmid loss in the *sld3-A dbf4-A* strain in the absence of genotoxic stress. Errors bars are SD, n=3.

E-G. 5 fold dilution growth assays of the indicated strains.

Supplemental Figure 7. Topological defects explains the DNA damage in the sld3-A dbf4-A strain

A and **B**. Quantification of Rad52-GFP foci in the indicated strains released from G1 phase arrest with alpha factor (0 mins) into 200mM HU. Error bars are SD, n=3.

C. Anti-HA western blot of HA-Top2 expressed from the GAL1-10 promoter in cells released from alpha factor (0) into 200mM HU in YPgalactose for the indicated time points. HA-Top2 protein is notably unstable under these conditions.

D. As A/B

E. Overlay of the flow cytometry profile from the indicated strains released from G1 phase into 0.033% MMS. As expected, the wild type strain has a slow S-phase in MMS due to checkpoint inhibition of origin firing, while the *sld3-A dbf4-A* strain completes S-phase progression (left overlay). The *sld3-A dbf4-A* strain that over-expresses HA-Top2 and Csm3/Tof1 also completes S-phase in MMS, indicating that this over-expression does not suppress the excess origin firing caused by the *sld3-A dbf4-A* alleles (right overlay).

F. Rad53 western blot of the experiment in Figure 7F/G. The indicated strains were released from G1 phase arrest with alpha factor (0 mins) into 200mM HU for 90 mins and then washed into HU-free media (release) for a further 150 minutes.
