# Supplementary material for: Checkpoint inhibition of origin firing prevents DNA topological stress
Source: Genes Dev. 2019 Nov 1;33(21-22):1539–54. doi: 10.1101/gad.328682.119 (PMC6824463; doi:10.1101/gad.328682.119)
Supplement: Supplemental Material [file supp_gad.328682.119_Supplemental_SuppTable4.docx]

**Supplemental­­­ Table S4.** *S. cerevisiae* strains and plasmids used in this study

All the strains used in this work are derived from W303 (*ade2-1 ura3-1 his3-11,15 trp1-1 leu2-3,112 can1-100, rad5-535)*

SLD3 and DBF4 mutants that cannot be phosphorylated by Rad53 (*sld3-38A and dbf4-4A*) are abbreviated to *sld3-A dbf4-A* in the main text.

| **Strain** | **Genotype** | **Source** |
| --- | --- | --- |
| yPZ 245 | *MATa RAD5^+^ exo1Δ::HIS3* | this work |
| yPZ 257 | *MATa RAD5^+^ rev3∆::LEU2* | this work |
| yPZ 264 | *MATα RAD5^+^ rad50∆::KanMx* | this work |
| yPZ 275 | *MATa RAD5^+^ rad52∆::LEU2* | this work |
| yPZ 277 | *MATa RAD5^+^ ku70∆::LEU2* | this work |
| yPZ 289 | *MATa RAD5^+^ rad52∆::LEU2 ku70∆::LEU2* | this work |
| yPZ 375 | *MATα RAD5^+^ sae2∆::KanMX* | this work |
| yPZ 445 | *MATa RAD5^+^ rnh201∆::HphMX* | this work |
| yPZ 705 | *MATa* | PZ lab |
| yPZ 917 | *MATa dbf4-4A::HIS3 sld3-38A-10his13myc ::KanmX* | PZ lab |
| yPZ 950 | *MATa hta1-S129A hta2-S129A* | Steve Jackson |
| yPZ 1302 | *MATa sml1-1 mec1Δ::TRP1 tel1Δ:::HIS3* | Steve Jackson |
| yPZ 1143 | *MATa sml1Δ::URA3 rad53Δ::LEU2 Rad52-eGFP::HphNT* | PZ lab |
| yPZ 1144 | *MATa Rad52-eGFP::HphNT dbf4-4A::HIS3 sld3-38A-10his13myc ::KanmX* | PZ lab |
| yPZ 1433 | *MATa* *csm3Δ::KanMx* | this work |
| yPZ 1446 | *MATa hta1-S129A hta2-S129A dbf4-4A::HIS3*  *sld3-38A-10his13myc::KanmX* | this work |
| yPZ 1447 | *MATa sml1-1 mec1Δ::TRP1 tel1Δ:::HIS3 dbf4-4A::HIS3 sld3-38A-10his13myc ::KanmX* | PZ lab |
| yPZ 1481 | *MATa ctf18Δ::URA3* | this work |
| yPZ 1483 | *MATa dbf4-4A::HIS3 sld3-38A-10his13myc::KanmX ctf18Δ::URA3* | this work |
| yPZ 1488 | *MATa RAD5^+^ rnh201∆::HphMX sld3-38A-10his13myc::KanmX dbf4-4A::HIS3* | this work |
| yPZ 1508 | *MATa* *csm3Δ::KanMX sld3-38A-10his13myc ::KanmX dbf4-4A::HIS3* | this work |
| yPZ 1510 | *MATa sgs1Δ::HPH dbf4-4A::HIS3 sld3-38A-10his13myc ::KanmX* | this work |
| yPZ 1513 | *MATa RAD5^+^ ku70∆::LEU2 sld3-38A-10his13myc::KanmX dbf4-4A::HIS3* | this work |
| yPZ 1525 | *MATa RAD5^+^ rev3∆::LEU2 sld3-38A-10his13myc::KanmX dbf4-4A::HIS3* | this work |
| yPZ 1544 | *MATa sgs1Δ::HPH* | PZ lab |
| yPZ 1552 | *MATa RAD5^+^ exo1Δ::HIS3 sld3-38A-10his13myc::KanmX dbf4-4A::HIS3* | this work |
| yPZ 1556 | *MATa ctf18Δ::URA3 top1Δ::NatMX* | this work |
| yPZ 1559 | *MATα dbf4-4A::HIS3 sld3-38A-10his13myc::KanmX ctf18Δ::URA3 top1Δ::NatMX* | this work |
| yPZ 1560 | *MATa bub3Δ::URA3* | this work |
| yPZ 1561 | *MATa RAD5^+^ sae2∆::KanMX sld3-38A-10his13myc::KanmX dbf4-4A::HIS3* | this work |
| yPZ 1563 | *MATa dbf4-4A::HIS3 sld3-38A-10his13myc ::KanmX bub3Δ::URA3* | this work |
| yPZ 1572 | *MATa RAD5^+^ rad52∆::LEU2 sld3-38A-10his13myc::KanmX dbf4-4A::HIS3* | this work |
| yPZ 1576 | *MATa mus81Δ::URA3* | this work |
| yPZ 1577 | *MATa dbf4-4A::HIS3 sld3-38A-10his13myc::KanmX mus81Δ::URA3* | this work |
| yPZ 1579 | *MATa tof1Δ::URA3* | PZ lab |
| yPZ 1580 | *MATa RAD5^+^ rad50∆::KanMX sld3-38A-10his13myc::KanmX dbf4-4A::HIS3* | this work |
| yPZ 1582 | *MATa dbf4-4A::HIS3 sld3-38A-10his13myc ::KanmX tof1Δ::URA3* | this work |
| yPZ 1738 | *MATa RAD5^+^ rad23∆::TRP1* | this work |
| yPZ 1743 | *MATa dbf4-4A::HIS3 sld3-38A-10his13myc ::KanmX tof1Δ::URA3 Rad52-eGFP::HphNT* | this work |
| yPZ 1744 | *MATa Rad52-eGFP::HphNT tof1Δ::URA3* | this work |
| yPZ 1776 | *MATα RAD5^+^ rad23∆::TRP1 sld3-38A-10his13myc::KanmX dbf4-4A::HIS3* | this work |
| yPZ 1808 | *MATa RAD5^+^ apn1∆::TRP1* | this work |
| yPZ 1819 | *MATa RAD5^+^ apn1∆::TRP1 dbf4-4A::HIS3 sld3-38A-10his13my ::KanmX* | this work |
| yPZ 1829 | *MATa trp1::SLD2-PGAL1-10-DPB11::TRP1 ura3::SLD3-PGAL1-10-DBF4::URA3 leu2::SLD7-PGAL1-10-CDC45::LEU2 Rad52-eGFP::HphNT* | this work |
| yPZ 1849 | *MATa RAD5^+^ msh2∆::TRP1* | this work |
| yPZ 1902 | *MATa RAD5^+^ msh2∆::TRP1 dbf4-4A::HIS3 sld3-38A-10his13my ::KanmX* | this work |
| yPZ 1912 | *MATa RAD5^+^ rad52∆::LEU2 ku70∆::LEU2 sld3-38A-10his13myc::KanmX dbf4-4A::HIS3* | this work |
| yPZ 1958 | *MATa Rad52-eGFP::HphNT* | PZ lab |
| yPZ 2077 | *MATa Rad52-eGFP::HphNT sml1Δ::KanMX* | this work |
| yPZ 2078 | *MATa Rad52-eGFP::HphNT sml1Δ::KanMX trp1::SLD2-PGAL1-10-DPB11::TRP1 ura3::SLD3-PGAL1-10-DBF4::URA3 leu2::SLD7-PGAL1-10-CDC45::LEU2* | this work |
| yPZ 2131 | *MATα UBR1::GAL1-10-Ubiquitin-M-LacI fragment-Myc-UBR1 (HIS3)*  *leu2-3::pCM244(CMVp-tetR’-SSN6, LEU2) x 3*  *top2-td TOP2 5’ upstream-100- to -1 replaced with kanMX-tTA (tetR-VP16)-tetO2 - Ub - DHFRts- Myc-linker)*  *tof1Δ::URA3* | this work |
| yPZ 2145 | *MATa UBR1::GAL1-10-Ubiquitin-M-LacI fragment-Myc-UBR1 (HIS3)*  *leu2-3::pCM244(CMVp-tetR’-SSN6, LEU2) x 3*  *top2-td TOP2 5’ upstream-100- to -1 replaced with kanMX-tTA (tetR-VP16)-tetO2 - Ub - DHFRts- Myc-linker)*  *dbf4-4A::HIS3*  *sld3-38A-10his13myc ::KanmX* | this work |
| yPZ 2753 | *MATa UBR1::GAL1-10-Ubiquitin-M-LacI fragment-Myc-UBR1 (HIS3)*  *leu2-3::pCM244(CMVp-tetR’-SSN6, LEU2) x 3*  *top2-td TOP2 5’ upstream-100- to -1 replaced with kanMX-tTA (tetR-VP16)-tetO2 - Ub - DHFRts- Myc-linker)*  *tof1Δ::URA3*  *sld3-38A-10his13myc ::KanmX*  *dbf4-4A::HIS3* | this work |
| yPZ 2263 | *MATa top1Δ::HphMX* | this work |
| yPZ 2265 | *MATa top1Δ::HphMX dbf4-4A::HIS3 sld3-38A-10his13myc::KanmX* | this work |
| yPZ 2435 | *MATa sml1Δ::KanMX* | this work |
| yPZ 2436 | *MATa sml1Δ::KanMX top2-4* | this work |
| yPZ 2547 | *MATa Rad52-eGFP::HphNT sml1Δ::KanMX dbf4-4A::HIS3 sld3-38A-10his13myc ::KanmX* | this work |
| yPZ 2718 | *MATa bub3Δ::URA3 top1Δ::NatMX* | this work |
| yPZ 2719 | *MATa bub3Δ::URA3 top1Δ::NatMX dbf4-4A::HIS3 sld3-38A-10his13myc::KanmX* | this work |
| yPZ 2721 | *MATa top1Δ::HphMX tof1Δ::URA3* | this work |
| yPZ 2723 | *MATa top1Δ::HphMX tof1Δ::URA3 dbf4-4A::HIS3 sld3-38A-10his13myc::KanmX* | this work |
| yPZ 2815 | *MATa top2-4 sml1Δ::KanMX trp1::SLD2-PGAL1-10-DPB11::TRP1 ura3::SLD3-PGAL1-10-DBF4::URA3 leu2::SLD7-PGAL1-10-CDC45::LEU2* | this work |
| yPZ 2830 | *MATa top1Δ::NatMX tof1Δ::URA3 Rad52-eGFP::HphNT* | this work |
| yPZ 2853 | *MATa csm3Δ::KanMx top1Δ::HphMX* | this work |
| yPZ 2856 | *MATa csm3Δ::KanMx top1Δ::HphMX dbf4-4A::HIS3 sld3-38A-10his13myc ::KanmX* | this work |
| yPZ 2871 | *MATa top1Δ::NatMX tof1Δ::URA3 Rad52-eGFP::HphNT*  *dbf4-4A::HIS3 sld3-38A-10his13myc ::KanmX* | this work |
| yPZ 2920 | *MATa mus81Δ::URA3 top1Δ::HphMX dbf4-4A::HIS3 sld3-38A-10his13myc::KanmX* | this work |
| yPZ 2945 | *MATa sgs1Δ::HPH top1Δ::HphMX dbf4-4A::HIS3 sld3-38A-10his13myc::KanmX* | this work |
| yPZ 2959 | *MATa Rad52-eGFP::HphNT Gal1-10-Csm3/Tof1::TRP1* | this work |
| yPZ 2960 | *MATa Rad52-eGFP::HphNT Gal1-10-Csm3/Tof1::TRP1*  *dbf4-4A::HIS3 sld3-38A-10his13myc::KanmX* | this work |
| yPZ 2964 | *MATa sgs1Δ::HPH top1Δ::HphMX* | this work |
| yPZ 2969 | *MATa Rad52-eGFP::HphNT Gal1-10-Top2::TRP1 dbf4-4A::HIS3 sld3-38A-10his13myc::KanmX* | this work |
| yPZ 2970 | *MATa Rad52-eGFP::HphNT Gal1-10-Top2::TRP1* | this work |
| yPZ 3110 | *MATa dbf4-4A::HIS3 sld3-38A-10his13myc ::KanmX mms4Δ::URA3 top1Δ::HphMX* | this work |
| yPZ 3121 | *MATa mus81Δ::URA3 top1Δ::HphMX* | this work |
| yPZ 3273 | *MATa ADE+ MYO1-GFP::HIS3 HTB2::mCherry::His5 mus81Δ::URA3 top1Δ::HphMX dbf4 4A::HIS3 sld3-38A-10his13myc::KanmX* | this work |
| yPZ 3275 | *MATa ADE+ MYO1-GFP::HIS3 HTB2::mCherry::His5 top1Δ::HphMX*  *dbf4-4A::HIS3 sld3-38A-10his13myc::KanmX* | this work |
| yPZ 3276 | *MATa ADE+ MYO1-GFP::HIS3 HTB2::mCherry::His5 dbf4-4A::HIS3 sld3-38A-10his13myc::KanmX* | this work |
| yPZ 3291 | *MATa ADE+ MYO1-GFP::HIS3 HTB2::mCherry::His5* | this work |
| yPZ 3292 | *MATa ADE+ MYO1-GFP::HIS3 HTB2::mCherry::His5 top1Δ::HphMX* | this work |
| yPZ 3310 | *MATa ADE+ MYO1-GFP::HIS3 HTB2::mCherry::His5 mus81Δ::URA3* | this work |
| yPZ 3311 | *MATa ADE+ MYO1-GFP::HIS3 HTB2::mCherry::His5 mus81Δ::URA3 top1Δ::HphMX* | this work |
| yPZ 3312 | *MATa ADE+ MYO1-GFP::HIS3 HTB2::mCherry::His5 mus81Δ::URA3 dbf4-4A::HIS3 sld3-38A-10his13myc ::KanmX* | this work |
| yPZ 3349 | *MATa top1Δ::HphMX tho2Δ::TRP1 dbf4-4A::HIS3 sld3-38A-10his13myc ::KanmX* | this work |
| yPZ 3364 | *MATa thp2Δ::TRP1* | this work |
| yPZ 3366 | *MATa tho2Δ::TRP1 dbf4-4A::HIS3 sld3-38A-10his13myc ::KanmX* | this work |
| yPZ 3368 | *MATa top1Δ::HphMX tho2Δ::TRP1* | this work |
| yPZ 3389 | *MATa top2-4 + pRS316* | this work |
| yPZ 3390 | *MATa top2-4 dbf4-4A::HIS3 sld3-38A-10his13myc ::KanmX + pRS316* | this work |
| yPZ 3578 | *MATa RAD5^+^* | PZ lab |
| yPZ 3581 | *MATa RAD5+ dbf4-4A::HIS3 sld3-38A-10his13myc::KanmX* | this work |
| yPZ 3706 | *MATa top2-4 + p1184 (10kb co-directional)* | this work |
| yPZ 3707 | *MATa top2-4 dbf4-4A::HIS3 sld3-38A-10his13myc ::KanmX + p1184 (10kb co-directional)* | this work |
| yPZ 3708 | *MATa top2-4 + p1185 (10kb convergent)* | this work |
| yPZ 3709 | *MATa top2-4 dbf4-4A::HIS3 sld3-38A-10his13myc ::KanmX + p1185 (10kb convergent)* | this work |
| yPZ 3806 | *MATα sml1Δ::KanMX trp1::SLD2-PGAL1-10-DPB11::TRP1 ura3::SLD3-PGAL1-10-DBF4::URA3 leu2::SLD7-PGAL1-10-CDC45::LEU2* | this work |

Notes:

*dbf4-4A* refers to the rad53 site mutant. It has serine/threonine to alanine mutations at amino acids: 518, 521, 526, 528.

*sld3-38A* contains the following S/T mutated to A;

306, 310, 421, 434, 435, 438, 442, 445 , 450, 451, 452, 456, 458, 459, 479, 482, 507 , 509, 514, 519,521,524, 540, 541, 546, 547, 548, 550, 556, 558, 559, 565, 569, 582, 607, 653 and 654. 539 is mutated to arginine.

*top2-4* is C2462A (Pro821 to Gln)

**Table of Plasmids**

| **N^o^** | **Plasmid description** | **Source** |
| --- | --- | --- |
| 86 | pRS316 | PZ lab |
| 809 | pARS1 CEN5 HygB | this work |
| 862 | pARS1 HygB Gal-ADE2 Convergent | this work |
| 863 | pARS1 HygB Gal-ADE2 Non-Convergent | this work |
| 1152 | pRS316 P_2tet-Cyc1_URA3 Co-directional (5 kb) | this work |
| 1153 | pRS316 P_2tet-Cyc1_URA3 Convergent (5 kb) | this work |
| 1184 | pRS316 P_2tet-Cyc1_URA3 Co-directional (10 kb) | this work |
| 1185 | pRS316 P_2tet-Cyc1_URA3 Convergent (10 kb) | this work |
